# Inhibition of Membrane-Bound BAFF by the Anti-BAFF Antibody Belimumab

**DOI:** 10.3389/fimmu.2018.02698

**Published:** 2018-11-20

**Authors:** Christine Kowalczyk-Quintas, Dehlia Chevalley, Laure Willen, Camilla Jandus, Michele Vigolo, Pascal Schneider

**Affiliations:** ^1^Department of Biochemistry, University of Lausanne, Lausanne, Switzerland; ^2^Department of Oncology UNIL CHUV, University of Lausanne, Lausanne, Switzerland

**Keywords:** BAFF, BLyS, furin, protein shedding, complement, antibody-dependent cell death

## Abstract

B cell activating factor of the TNF family (BAFF, also known as BLyS), a cytokine that regulates homeostasis of peripheral B cells, is elevated in the circulation of patients with autoimmune diseases such as systemic lupus erythematosus (SLE). BAFF is synthetized as a membrane-bound protein that can be processed to a soluble form after cleavage at a furin consensus sequence, a site that in principle can be recognized by any of the several proteases of the pro-protein convertase family. Belimumab is a human antibody approved for the treatment of SLE, often cited as specific for the soluble form of BAFF. Here we show in different experimental systems, including in a monocytic cell line (U937) that naturally expresses BAFF, that belimumab binds to membrane-bound BAFF with similar EC50 as the positive control atacicept, which is a decoy receptor for both BAFF and the related cytokine APRIL (a proliferation inducing ligand). In U937 cells, binding of both reagents was only detectable in furin-deficient U937 cells, showing that furin is the main BAFF processing protease in these cells. In CHO cells expressing membrane-bound BAFF lacking the stalk region, belimumab inhibited the activity of membrane-bound BAFF less efficiently than atacicept, while in furin-deficient U937 cells, belimumab inhibited membrane-bound BAFF and residual soluble BAFF as efficiently as atacicept. These reagents did not activate complement or antibody-dependent cell cytotoxicity upon binding to membrane-bound BAFF *in vitro*. In conclusion, our data show that belimumab can inhibit membrane-bound BAFF, and that BAFF in U937 cells is processed by furin.

## Introduction

BAFF and APRIL are important fitness and survival factors for B cells and plasma cells in the periphery. They exert their function through different receptors: BAFFR (BAFF receptor, TNFRSF13A) that binds to BAFF only, TACI (transmembrane activator and calcium modulator and cyclophilin ligand interactor, TNFRSF13B) that binds to BAFF and APRIL, and BCMA (B cell maturation antigen, TNFRSF17) that also binds to BAFF and APRIL [reviewed in (1)]. BAFFR transduces BAFF survival signals in transitional and naïve B cells, both of which are greatly decreased in BAFF-ko and BAFFR-ko mice (2–5). TACI and BCMA are expressed either upon B cell activation and/or at later stages of B cell differentiation. For example, BCMA is expressed in plasma cells that can use APRIL and/or BAFF for survival (6). Although BAFF is synthetized as a membrane-bound protein, it can be processed to a soluble form by cleavage at a furin consensus-processing site (7, 8). Furin belongs to the substilisin/kexin-like pro-protein convertase (PCSK) family of proteases, seven of which (PCSK1-2, furin and PCSK4-7) have arginines in their recognition sequences, and most of which are ubiquitously expressed. They are often redundant for substrate cleavage and they process a vast panel of targets, among others hormones, enzymes, receptors, cytokines and extracellular matrix components [reviewed in (9)]. With regard to BAFF, circulating levels are found elevated in diseases with involvement of auto-reactive B cells, including systemic lupus erythematosus (SLE) [reviewed in (10, 11)]. Belimumab, a human monoclonal IgG1λ anti-BAFF antibody approved by the FDA, can improve the condition of SLE patients (12, 13). Belimumab is specific for BAFF, and is more precisely described as an inhibitor of soluble BAFF (14, 15). Atacicept is another BAFF inhibitor consisting of the ligand-binding portion of the receptor TACI fused to a modified Fc portion of human IgG1 to remove binding to Fc receptors and to complement. Atacicept is characterized by a broader specificity of inhibition that includes APRIL and heteromers of BAFF and APRIL (16). Atacicept is under clinical development, also for the treatment of SLE (17). Here, we genetically inactivated furin in U937 histiocytic lymphoma cells that naturally express BAFF (18) to convert these cells form BAFF shedding into membrane-bound BAFF-expressing cells, indicating that furin itself is the main BAFF-processing protease in these cells. Membrane-bound BAFF on furin-deficient U937 was bound and inhibited by belimumab, suggesting that belimumab targets membrane-bound in addition to soluble BAFF.

## Materials and methods

### Proteins and antibodies

Belimumab (registered trade name Benlysta), denosumab (registered trade name Xgeva), adalimumab (registered trade name Humira), and human IgG (intraveinous immunoglobulins; registered trade name Kiovig) were purchased from the Pharmacy of Lausanne University Hospital (CHUV). Atacicept was kindly provided by Henry Hess (Merck KGaA). BCMA-Fc and Fc-BAFF were produced in CHO cells and affinity purified on Protein A-Sepharose, essentially as described (19). When indicated, dimeric BCMA-Fc was used. Dimeric BCMA-Fc was obtained as a defined peak after size fractionated by gel filtration on a Superdex 200 Increase 10/30 column (GE Healthcare) equilibrated in PBS. BCMA-COMP-Flag (20) was produced by transient transfection of HEK 293T cells with the polyethyleneimide method (21). 7-day-old conditioned cell supernatants in serum-free OptiMEM medium were collected and used directly.

### Cell lines

HEK 293T and histiocytic lymphoma U937 cells were obtained form late Jürg Tschopp (University of Lausanne). CHO-S cells were from Thermoscientific (A1155701). CHO-3296 clone 7 expressing uncleavable BAFF (hBAFF Δ85-136) was obtained by transfection with polyethyleneimide, selection for 3 passages in 500 μg/ml of G418 sulfate (Calbiochem, 345812) and cloning by limiting dilution. Clones were screened for BAFF expression by staining with atacicept by flow cytometry (see “Flow cytometry”). U937 cell deficient for BAFF (U937-3515 clone B9) and U937 cells deficient for furin (U937-3511 clone D3) were generated by lentiviral transduction of CRISPR/Cas9-expression vectors with hBAFF gRNA, respectively hFurin gRNA (Supplementary Table 1). Annealed oligonucleotides 5′- CACCGACTGATAAGACCTACGCCAT-3′ and 5′- AAACATGGCCGTAGGTCTTATCAGT-3′ (for hBAFF) or 5′- CACCGAAGTGCACGGAGTCTCACAC-3′ and 5′- AAACGTGTGAGACTCCGTGCACTTC-3′ (for hFurin) were cloned in the BsmBI restriction site of lentrcrispr v2 plasmid (Addgene #52961) (22). These plasmids were co-transfected with co-vectors pCMV-VSV-g (Addgene #8454) and psPAX2 (Addgene #12260) (Supplementary Table 1) into 293T cells with polyethyleneimide. The next day, cells were washed with PBS and cultured for an additional day in RPMI 10% FCS. Cell supernatants filtered at 0.45 μm were supplemented with 8 μg/ml polybrene (Sigma, H9268) and 2.5 ml were added to 2 × 10^6^ pelleted U937 cells that were subsequently cultured overnight in a 12-well plate, then washed and expanded for 3 days in a 6-well plate in RPMI 10% FCS. Cells were then selected for 3 days in RPMI 10% FCS, 1 μg/ml puromycin (EnzoLifeSciences ALX 380-028). Surviving cells were cloned in RPMI 10% FCS, in culture plates pre-coated for 5 min at 37°C with 1 μg/ml of poly-lysine (Sigma P6407) in water. Furin-deficient clones and BAFF-deficient clones were identified by their impairment to release active soluble BAFF in their supernatants (see “Cytotoxic assays”). Jurkat JOM2 BAFFR:Fas-2308 cl21 reporter cells were as described (16, 23, 24). Jurkat cells expressing FcγRIIIa and NFAT-driven luciferase were provided in the ADCC reporter bioassay core kit (Promega, G7010) and used as recommended by the manufacturer.

### Biotinylations

One milligram of belimumab or 1 mg of atacicept in 1 ml of 0.1 M Na-borate pH 8.8 were biotinylated for 2 h at room temperature with 10 μl of EZ-Link^Tm^ sulfo-N-hydroxysuccinimide-LC-biotin (Pierce, #21335) at 30 μg/ml in DMSO. Reaction was terminated by addition of 30 μl of 1 M NH_4_Cl, and buffer was exchanged to PBS using a 30 kDa cutoff centrifugal device.

### CFSE labeling

Carboxyfluorescein-succinimidyl ester (CFSE, Sigma 21888) at 10 mM in dimethylsulfoxide was stored at −70°C until use. BAFFR:Fas reporter cells (~10^7^) were labeled by incubation for 8 min at 37°C in a freshly prepared mixture of PBS, 1% FCS and 2 μM CFSE, or in PBS, 0.1 μM CFSE. Reactions were quenched by the addition of RPMI 10% FCS, cells were harvested, washed twice with RPMI 10% FCS and used to assay membrane-bound BAFF (see “Cytotoxic assays with BAFFR:Fas reporter cells”).

### Cytotoxic assays with BAFFR:Fas reporter cells

Recombinant Fc-hBAFF was used at the indicated concentrations, and BAFF in conditioned medium of CHO or U937 cells (parental or stable clones) was used at the indicated dilutions of supernatants. Inhibitors, when present, were used at the indicated concentrations and pre-incubated for at least 3 min with effectors in 50 μl of RPMI 10% FCS. Unlabeled Jurkat JOM2 BAFFR:Fas-2308 clone 21 reporter cells (20,000–50,000/well) were added in 50 μl of RPMI 10% FCS and incubated overnight (~16 h) at 37°C, 5% CO_2_. Cell viability was then recorded by addition of 20 μl of PMS/MTS (phenazine methosulfate at 45 μg/ml and 3-(4,5-dimethylthiazol-2-yl)-5-(3-carboxymethoxyphenyl)-2-(4-sulfophenyl)-2H-tetrazolium at 2 mg/ml in PBS) and by monitoring absorbance at 492 nm after adequate color development (~2–8 h) (24).

The measure of membrane-bound BAFF activity on U937 cells was performed in 96 well plates, in a final volume of 100 μl of RPMI 10% FCS, at 37°C and with 5% CO_2_. 50 μl of effector cells (U937-3515 clone B9 or U937-3511 clone D3, 10^5^ cells), or 50 μl of soluble effectors (Fc-hBAFF at 100 ng/ml) were mixed with 20 μl of 5-fold concentrated inhibitors for 6 h, then 30 μl of CFSE-labeled BAFFR:Fas reporter cells (~5 × 10^4^) were added for an additional 11 h. Cells were transferred on ice in 1 ml tubes containing 10 μl of 10 μg/ml propidium iodide, and analyzed by flow cytometry (See “Flow cytometry”).

For the measure of membrane-bound BAFF on CHO-3296 cl7 cells, experiments were performed in 48 well plates. Untransfected CHO cells, or CHO-3296 cl7 cells (1.5 × 10^5^/well) were left to adhere for 2 h, after which time medium was removed and replaced by 200 μl of fresh medium. Wells without CHO cells contained medium only. Hundred μl of inhibitors (at 5-fold the final desired concentration), 50 μl of Fc-BAFF at 1 μg/ml (or 50 μl of medium in conditions without Fc-BAFF) and 150 μl of CFSE-labeled BAFFR:Fas reporter cells (~2.5 × 10^5^ cells) were added and incubated for 11 h. Non adherent cells were transferred into 4 ml tubes, and then pooled with adherent cells detached with trypsin-EDTA. Cells were spun for 1 min at 5,000 rpm (2,400 × *g*), pellets were suspended in 100 μl of PBS, 5% FCS and supplemented with 10 μl of 10 μg/ml propidium iodide before analysis (See “Flow cytometry”).

### Flow cytometry

293T cells were co-transfected with hBAFF expression vectors and an EGFP expression vector as a tracer. Cells were stained 3 days later with the indicated amounts of atacicept or belimumab in 50 μl of PBS, 5% FCS for 20 min on ice, followed by phycoerythrin-coupled rat anti-human IgG (Southern Biotech Associate, 2040-09, 1/500). Alternatively, transfected cells were pre-incubated with 5 μg of atacicept or belimumab in 25 μl of PBS 5% FCS for 20 min on ice, then stained for 20 min, without wash, by addition of 25 μl of BCMA-COMP-Flag in conditioned OptiMEM supernatants, followed by biotinylated anti-FLAG (Sigma, F9291, 1/500) and phycoerythrin-coupled streptavidin (eBioSciences, 12-4317-87, 1/500). Data was acquired with an AccuriC6 flow cytometer (BD Bioscience) and analyzed with the FlowJo software (TreeStar, Ashland, OR). Mean fluorescence intensity of atacicept, belimumab or BCMA-COMP-Flag staining was measured for cells expressing medium levels (2 × 10^4^ to 2 × 10^5^ fluorescence units) of EGFP.

CHO-3296 clone 7 cells were stained with atacicept, belimumab or denosumab as described for transfected 293T cells, except that MFI was reported for all live cells. U937 cells (wild type, furin-deficient or BAFF-deficient) were pre-incubated with human IVIGs (50 μg in 50 μl of PBS, 5% FCS) for 20 min on ice, washed and stained for 20 min on ice with the indicated amount of biotinylated atacicept or biotinylated belimumab in 50 μl of PBS 5% FCS followed by phycoerythrin-coupled streptavidin (1/500).

CFSE-labeled reporter cells (co-cultured with membrane-bound BAFF-expressing cells, recombinant Fc-BAFF or controls) were stained with propidium iodide as described (see “Cytotoxic assays with BAFFR:Fas reporter cells”) and analyzed by flow cytometry. The proportion of live reporter cells was calculated as follows: [number of live reporter cells / (number of live reporter cells + number of dead reporter cells)], where live cells were CFSE^+^ PI^−^ cells in the live FCS/SSC gate, and dead cells were all CFSE^+^ cells in the dead FCS/SSC gate. This number was divided by the proportion of live cells in untreated controls to correct for spontaneous death, and multiplied by 100 to get the percentage of specific target cell viability.

### BAFF ELISA

BAFF in conditioned supernatant of U937 cells was quantified using the hypersensitive hBAFF ELISA kit (Adipogen, AG-45B-0001-KI01) according to the manufacturer's protocol. Supernatants of BAFF-deficient and furin-deficient cells were measured in duplicate at a dilution of 1/2, and those of wild type cells at dilutions of 1/4, 1/40, and 1/400.

### Complement activation assay

CHO-3296 clone 7 cells or CHO cells (2 × 10^4^/well in 80 μl of DMEM/F12 (1/1) 2% FCS) were left to adhere overnight in 96-wells culture plates. Fc-containing reagents were then added in 10 μl of medium, incubated for 10 min at room temperature before addition of 10 μl of untreated or heat-inactivated (30 min at 56°C) normal human serum (from P.S.) for 2 h at 37°C. Cell viability was monitored with PMS/MTS (see “Cytotoxic assays with BAFFR:Fas reporter cells”).

### Antibody-dependent cell-mediated cytotoxicity assay

Antibody-dependent cell-mediated cytotoxicity was measured using the ADCC core kit (Promega, G7010), according to the manufacturer's protocol. CHO-3296 clone 7 cells were used as targets, adalimumab, atacicept, BCMA-Fc and belimumab at the indicated concentrations were used as mediators, and Jurkat cells expressing both human FcγRIIIa V158 and NFAT-induced luciferase were used as effectors. 25,000 target cells per well were seeded in 96 wells white plates in 100 μl of DMEM:F12 2% FCS and left to adhere overnight. Ninety-five microliter of medium was removed and replaced by 25 μl of RPMI supplemented with 4% of low Ig FCS, 25 μl of the test reagent and 25 μl of freshly defrosted effector cells. After 6 h of incubation, 75 μl of luciferase substrate was added and luminescence was monitored 15 min later.

### Statistics

Replicate measurements are shown as mean ± SEM. EC_50_ of titration curves were determined after normalization of cell viability using the “non linear regression (curve fit)” followed by the “log(agonist) vs. normalized response–variable slope” function of Prism 7 (GraphPad Software).

## Results

### Belimumab and atacicept bind to and inhibit BAFF at the surface of transfected 293T or CHO cells

**Figure 1:**
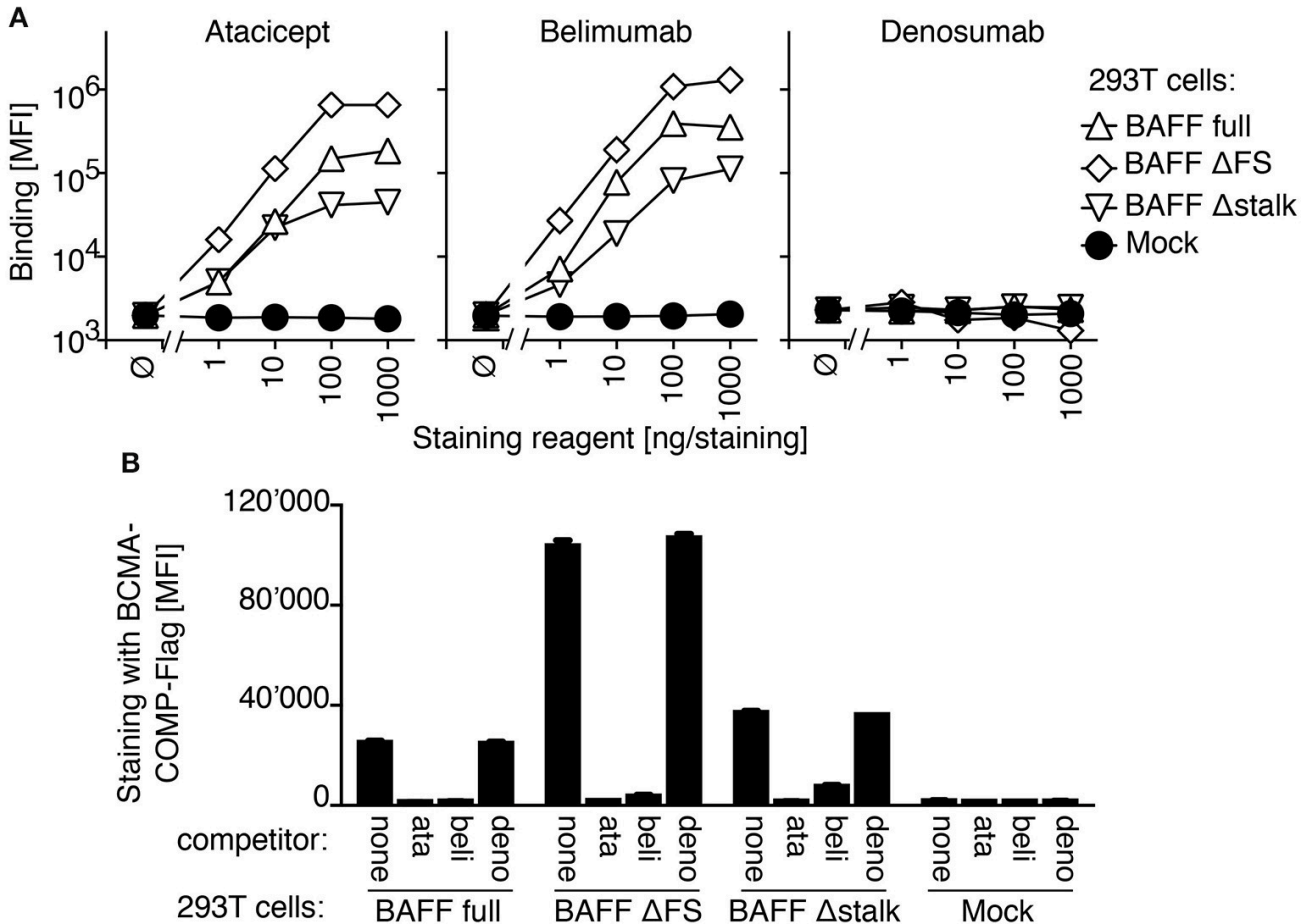
Belimumab binding to BAFF on transfected 293T cells prevents concomitant binding of BCMA. **(A)** 293T cells transiently transfected with plasmids coding for full length BAFF wild type (BAFF full), full length BAFF with mutated furin site (BAFF ΔFS), full length BAFF deleted for the stalk region and furin site (BAFF Δstalk) or with empty plasmid (Mock) were stained with the indicated amounts of belimumab or atacicept, or denosumab as control. Mean fluorescence intensity (MFI) of staining (in the phycoerythrin chanel) was monitored for cells expressing medium levels of co-transfected EGFP (2 × 10^5^ to 6 × 10^5^ fluorescence units). Mean ± SEM of triplicate measures (note that error bars are smaller than symbols). Experiment performed 5 times (twice in monoplicates, without BAFF Δstalk and denosumab controls). **(B)** 293T cells transfected as indicated (transfection distinct from that of panel **A**) were pre-incubated with buffer (none), atacicept (ata), belimumab (beli) or denosumab (deno) and then stained with BCMA-COMP-Flag and anti-Flag secondary reagents. Mean fluorescence intensity (MFI) was measured as in panel **A**. Mean ± SEM of triplicate measures. Experiment performed 3 times (once in monoplicate, without denosumab control).

In a FACS-based assay, both atacicept and belimumab stained 293T cells transfected with full length BAFF, but not mock-transfected cells. BAFF with a mutated furin consensus sequence (SRNKRAVGP → SRNKLQGP) (BAFF ΔFS) or BAFF lacking the stalk region linking the transmembrane domain to the extracellular receptor-binding domain, a deletion that included the furin consensus sequence (BAFF Δstalk) were also recognized by atacicept and belimumab, while the unrelated antibody denosumab stained none of these cells (Figure 1A). Binding of atacicept or belimumab to membrane-bound BAFF prevented subsequent binding of recombinant BCMA, one of the receptors for BAFF, suggesting that the binding of either atacicept or belimumab inhibits membrane-bound BAFF (Figure 1B).

**Figure 2:**
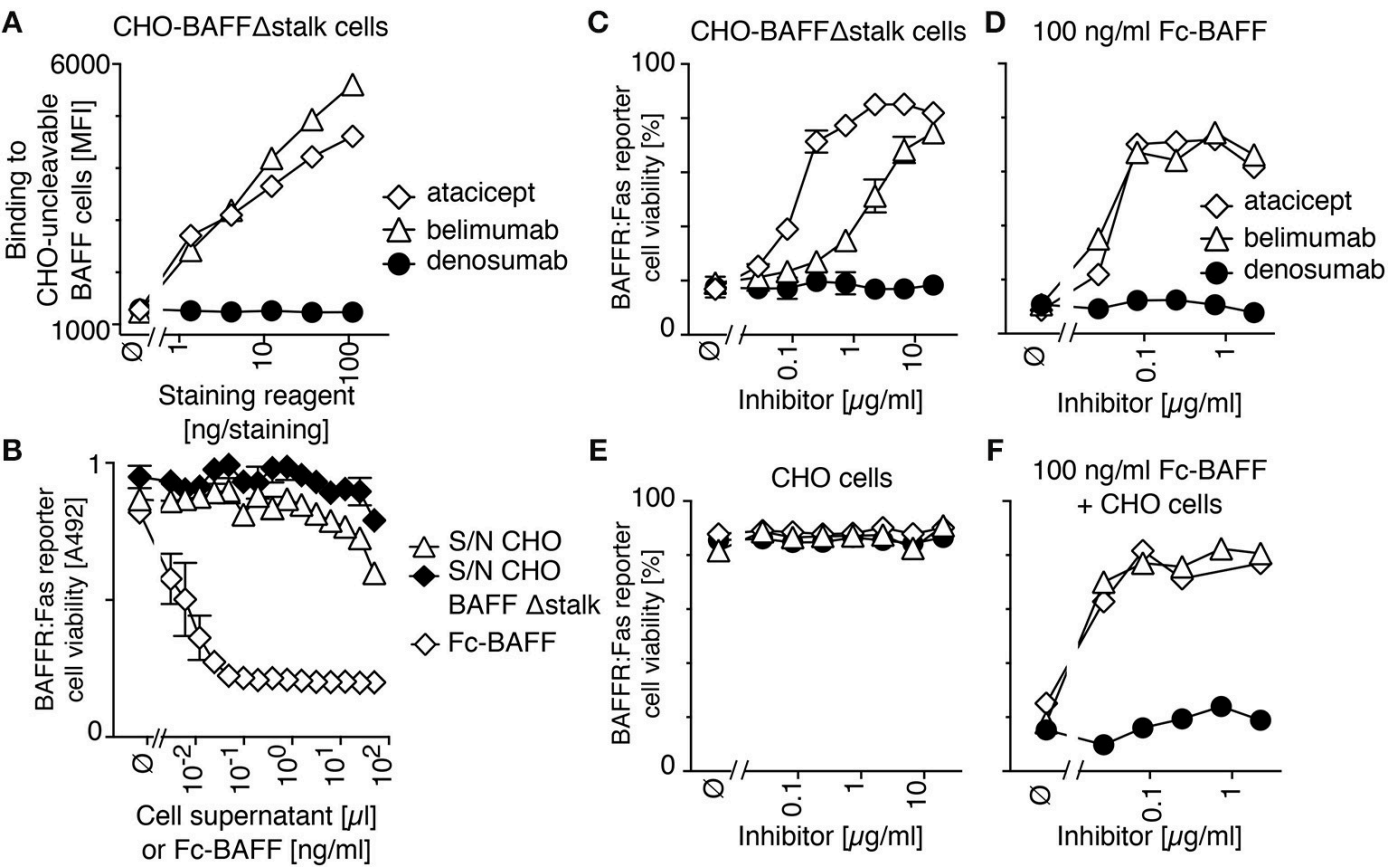
Belimumab binds and partially inhibits membrane-bound BAFF lacking the stalk region expressed in CHO cells. **(A)** CHO cells stably expressing uncleavable BAFF Δstalk were stained with the indicated amounts of atacicept, belimumab or denosumab. Mean fluorescence intensity (MFI) of staining was monitored. Single measures were performed. Experiment performed 3 times. **(B)** BAFFR:Fas reporter cells were exposed to titrated amounts of conditioned supernatants of CHO cells with or without expression of uncleavable BAFF Δstalk, or to recombinant soluble BAFF (Fc-BAFF) added as a positive control. After overnight incubation, cell viability was monitored with the PMS/MTS test. Mean ± SEM of triplicates. Experiment performed twice. **(C)** CFSE-labeled BAFFR:Fas reporter cells were co-cultured with CHO cells expressing uncleavable BAFF in the presence of increasing concentrations of atacicept, belimumab or denosumab. Cell viability after overnight incubation was measured by flow cytometry. Measures are duplicates (mean ± SEM). Experiment performed twice. **(D)** Same as panel **C**, except that uncleavable BAFF Δstalk cells were replaced by a lethal dose of Fc-BAFF (100 ng/ml). Single measures were performed. Experiment performed twice. **(E)** Same as panel C, except that uncleavable BAFF Δstalk cells were replaced by control CHO cells. Single measures were performed. Experiment performed twice. **(F)** CFSE-labeled BAFFR:Fas reporter cells were co-cultured overnight with CHO cells and 100 ng/ml of Fc-BAFF, in the presence of inhibitors at the indicated concentrations. Single measures were performed. Experiment performed twice.

In order to measure the activity of membrane-bound BAFF, we generated a stable clone of CHO cells expressing BAFF Δstalk, i.e., uncleavable membrane-bound BAFF. These cells stained with both atacicept and belimumab, but not with the control antibody denosumab (anti-RANKL) (Figure 2A) and did not release detectable levels of soluble BAFF activity in their supernatants (Figure 2B). Unlabeled CHO cells expressing uncleavable BAFF were co-cultured with CFSE-labeled BAFFR:Fas reporter cells. In reporter cells, the extracellular domain of BAFFR is fused to the transmembrane and intracellular domains of the death receptor Fas, so that they are killed upon engagement of BAFFR, and so that BAFF inhibitors can protect them from BAFF-mediated killing. CHO cells expressing uncleavable BAFF readily killed BAFFR:Fas reporter cells, a killing that could be inhibited in a dose-dependent manner by both atacicept and belimumab, although atacicept was about 10 times more efficient than belimumab in this respect (Figure 2C). Reporter cells also died in response to soluble recombinant BAFF, but in this case inhibition by atacicept or belimumab was equally good (Figure 2D). Controls showed that atacicept, belimumab or untransfected CHO cells had no deleterious effect on reporter cells (Figure 2E), and that untransfected CHO cells did not prevent atacicept and belimumab from inhibiting soluble BAFF (Figure 2F). Taken together, these results show that binding of atacicept and belimumab to membrane-bound BAFF is inhibitory.

### Processing of membrane-bound BAFF to active soluble BAFF is mediated by furin in U937 cells

**Figure 3:**
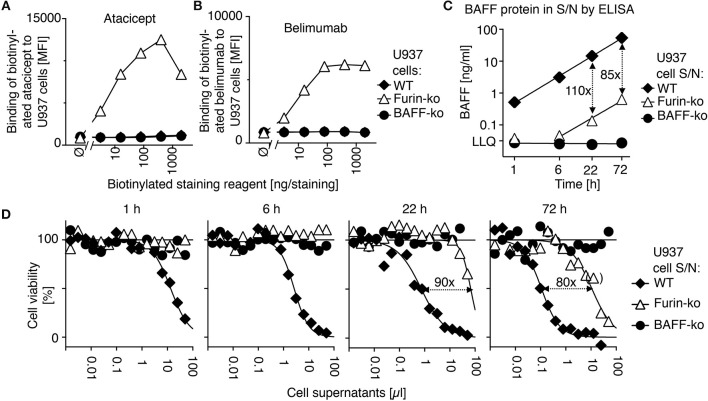
Belimumab binds to membrane-bound BAFF in furin-deficient U937 cells. **(A)** Wild-type (WT), furin-deficient (furin-ko) and BAFF-deficient (BAFF-ko) U937 cells were stained with the indicated amounts of biotinylated atacicept after saturation of Fc receptors with human IgGs. Mean fluorescence intensity (MFI) of staining was monitored. Single measures were performed. Experiment performed four times. **(B)** Same as panel A, except that staining was performed with biotinylated belimumab. Experiment performed four times. **(C)** The indicated U937 cells were washed and then put in culture. Human BAFF was quantified by ELISA in conditioned cell supernatants collected at the indicated time points. LLQ: lower limit of quantification. Double-sided arrows indicate the fold difference between concentrations of BAFF in supernatants of wild type and furin-deficient U937 cells. Error bars of measures performed in duplicate (mean ± SEM) are smaller than symbol size. Experiment performed twice. **(D)** BAFFR:Fas reporter cells were exposed to titrated amounts of the same conditioned supernatants used in panel C. After an overnight incubation, cell viability was monitored with the PMS/MTS assay. Single measures were performed. Double sided arrows indicate the fold difference between EC_50_ of wild type and furin-deficient supernatants. One measure indicated in brackets was excluded for the determination of EC_50_. Experiment performed twice in this format, and two more times with a single time point.

In order to determine the action of atacicept and belimumab on endogenous BAFF, we used monocytic U937 cells that naturally produce soluble BAFF (18, 25). Atacicept and belimumab did not stain wild type and BAFF-deficient U937 cells by flow cytometry (Figures 3A,B). However, CRISPR/Cas9-mediated deletion of furin with a construct targeting the active site of the protease abrogated >98% of the release of both BAFF protein (Figure 3C) and BAFF activity (Figure 3D) in supernatants of U937 cells, while increasing cell surface levels of BAFF that became detectable by staining with both atacicept and belimumab (Figures 3A,B).

### Inhibition of membrane-bound BAFF in U937 cells by atacicept and belimumab

**Figure 4:**
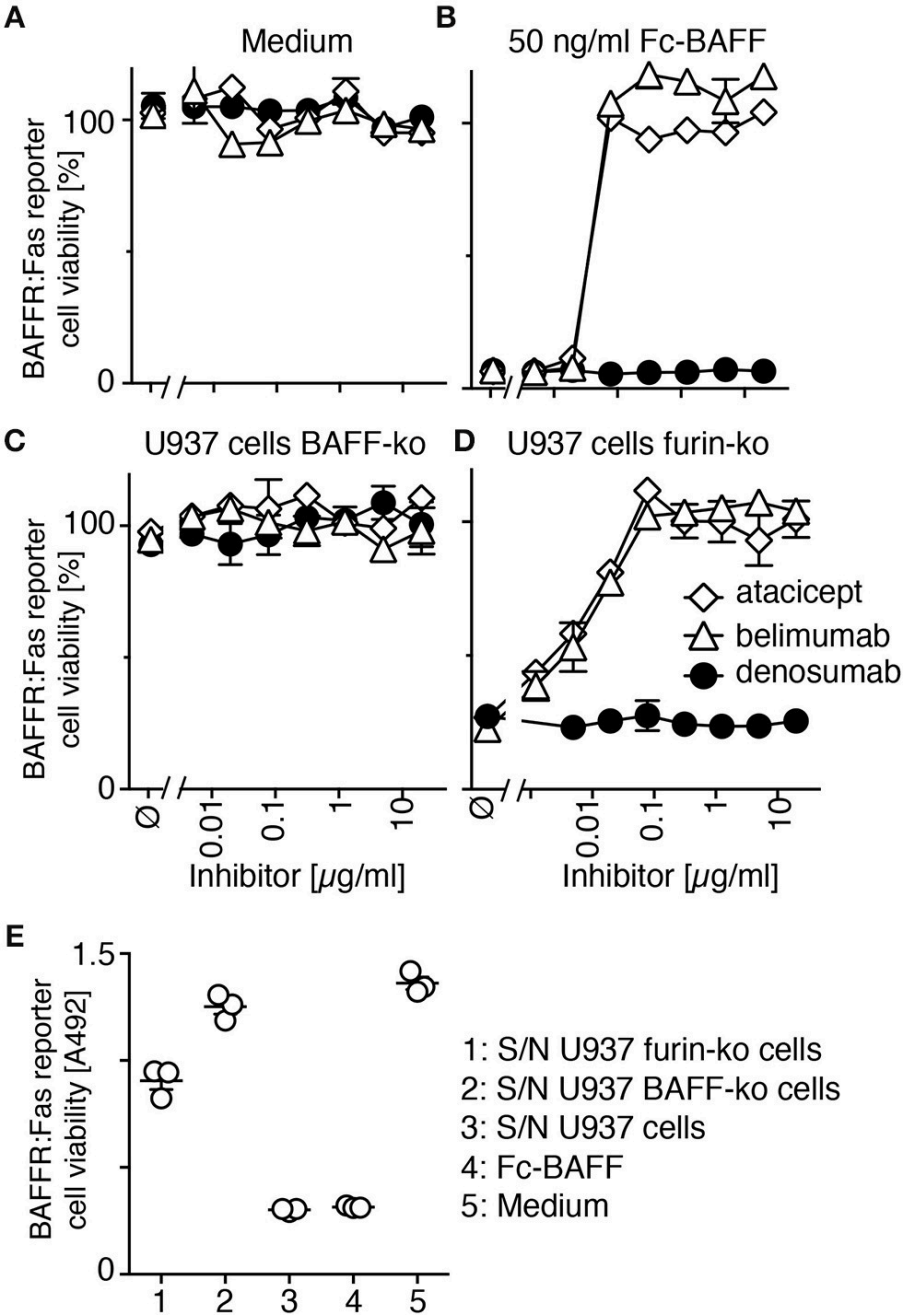
Belimumab inhibits membrane-bound and residual soluble BAFF in furin-deficient U937 cells. **(A,B)** CFSE-labeled BAFFR:Fas reporter cells were exposed to **(A)** medium or to **(B)** a lethal dose of Fc-BAFF (50 ng/ml) in the presence of increasing concentrations of atacicept, belimumab or denosumab. Cell viability after overnight incubation was measured by flow cytometry. Mean of triplicates ± SEM. Experiment performed twice. **(C,D)** CFSE-labeled BAFFR:Fas reporter cells were co-cultured overnight with **(C)** BAFF-deficient (BAFF-ko) or **(D)** furin-deficient (furin-ko) U937 cells in the presence of atacicept, belimumab or denosumab at the indicated concentrations. Mean of triplicates ± SEM. Experiment performed twice. **(E)** BAFFR:Fas reporter cells were exposed to undiluted conditioned medium of the indicated U937 cells that were grown alone, but for the same time (17 h) and otherwise identical conditions to those of U937 cells used in panels **C** and **D**. Medium and Fc-BAFF at 50 ng/ml in medium pre-incubated alone for 17 h in the same conditions were used as controls. Viability of reporter cells was monitored with the PMS/MTS assay. Mean of triplicates ± SEM. Experiment performed twice.

Inhibition of non-mutated membrane-bound BAFF was tested in furin-deleted U937 cells. Atacicept and belimumab on their own were not toxic for reporter cells (Figure 4A). On the contrary, they both protected reporter cells from a lethal dose of soluble recombinant BAFF at roughly stoichiometric ratio (Figure 4B). BAFF-deficient U937 cells were harmless to BAFFR:Fas reporter cells (Figure 4C), but furin-deficient U937 cells, which express membrane-bound BAFF, efficiently killed them (Figure 4D). This killing was inhibited in a dose-dependent manner and with similar efficacy by both atacicept and belimumab, while the control antibody denosumab did not protect against membrane-bound BAFF (Figure 4D). An additional control showed that BAFF released into supernatant of furin-deficient U937 cells during the 17 h co-culture period could only kill about 30% of reporter cells (Figure 4E), indicating that the remaining toxicity of furin-deficient U937 cells on BAFFR:Fas reporter cells was due to membrane-bound BAFF (Figure 4D). Taken together, these results indicate that both endogenous membrane-bound BAFF and residual soluble BAFF in U937 cells are bound and inhibited by atacicept and belimumab.

### Opsonized atacicept and belimumab do not induce complement or cell-mediated toxicity

**Figure 5:**
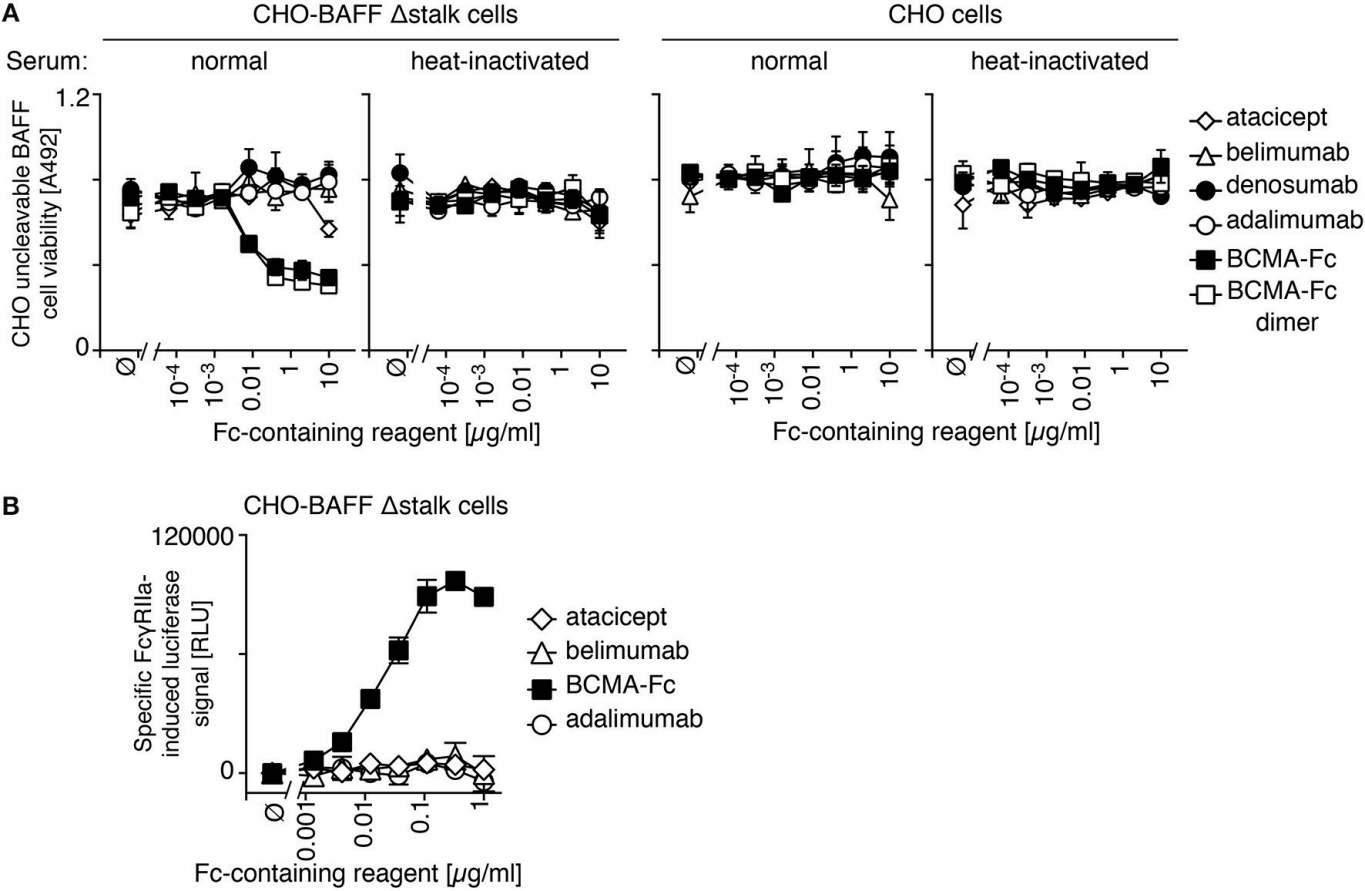
No detectable effector function of belimumab bound to membrane-bound BAFF. **(A)** CHO BAFF Δstalk cells or CHO cells were pre-incubated for 30 min with atacicept, belimumab, denosumab, adalimumab, BCMA-Fc or size-fractionated (dimeric) BCMA-Fc at the indicated concentrations, and subsequently exposed for 2 h to normal human serum that had been heat-inactivated or not. Cells viability was monitored with the PMS/MTS assay. Mean ± SEM of duplicates. Experiment performed twice (but only once for the condition of dimeric BCMA-Fc). **(B)** CHO BAFF Δstalk cells were pre-incubated with atacicept, belimumab, BCMA-Fc, or adalimumab at the indicated concentrations, after which time surrogate effector cells of antibody-dependent cellular-mediated cytotoxicity were added. NFAT-driven luciferase expression is induced when opsonized IgG-containing molecules engage FcγRIIIa expressed in these reporter cells. Specific signal in relative luminescence units (RLU) was obtained after subtraction of background of unstimulated cells (~10^5^ RLU). Mean ± SEM of triplicate measures. Experiment performed twice.

IgGs that are opsonized on a cell surface activate complement or antibody-dependent cell-mediated cytotoxicity via their fragment crystallisable (Fc) regions. Atacicept was used as a negative control because its mutated Fc region activates neither of these pathways. Neither atacicept nor belimumab activated complement when bound to CHO cells expressing uncleavable BAFF (Figure 5A). In contrast, BCMA-Fc, a BAFF-binding decoy receptor with a wild type human IgG1 Fc portion, decreased viability of target cells in the presence of normal human serum, but not in de-complemented serum and not in CHO cells expressing no BAFF (Figure 5A). Adalimumab, an anti-TNF antibody with reported ability to activate complement (26) had no activity in the absence of its target (Figure 5A). It is noteworthy that a purely dimeric preparation of BCMA-Fc, obtained after gel filtration, displayed the same activity as “crude” BCMA-Fc (Figure 5A).

Antibody-dependent cell-mediated cytotoxicity was monitored in a surrogate assay in which the activation of Fcγ receptor IIIa over-expressed in Jurkat T cells drives NFAT (nuclear factor of activated T cells)-dependent expression of luciferase. Neither atacicept nor belimumab activated reporter cells when bound to membrane-bound BAFF on CHO cells, while the positive control BCMA-Fc did (Figure 5B). We conclude from these data that atacicept and belimumab inhibit their target cytokine(s) in soluble or membrane-bound forms, but do not kill BAFF-expressing cells, at least *in vitro*.

## Discussion

It was previously reported that belimumab inhibits BAFF, but not APRIL. In addition, belimumab does not inhibit any of the BAFF-APRIL heteromers, while atacicept inhibits BAFF, APRIL and heteromers thereof (16). Another difference is that soluble BAFF can exist as 3-mers, or can assemble as 60-mers (27). Belimumab cannot inhibit BAFF 60-mers unless they first dissociate into 3-mers, whereas atacicept and another anti-BAFF antibody, tabalumab, can inhibit BAFF 60-mers (28–30). Belimumab is often described in the literature as an inhibitor of soluble BAFF. This conclusion probably finds its origin into the original description of the antibody, where belimumab could not stain IFNγ-stimulated human monocytes by flow cytometry, while another antibody called 12D6 could (14). This was later interpreted as “ […], belimumab specifically recognizes the soluble, biologically active form of BLyS” (31). Our present results question this conclusion and re-enforce previous results obtained with over-expressed BAFF in CHO cells showing that belimumab can inhibit membrane-bound BAFF (28). The monoclonal antibodies 12D6, 2E5, and 9B6 can all stain human monocytes (7, 14, 18). It would be interesting to monitor their binding to wild type, furin-deficient and BAFF-deficient U937 cells side by side with belimumab to determine if they are really better binders of membrane-bound BAFF, or whether there might be other causes for their binding to monocytes.

It has long been recognized that BAFF is processed at a furin consensus site (7, 8). Here we provide the first indication that in U937 cells, BAFF is cleaved by furin, with no or little contribution of other members of the pro-protein convertase subtilisin/kexin family. However, we did not investigate whether this is also true for other cell lines or primary cells. Like *Baff*-ko mice, mice with non-cleavable BAFF have few mature B cells, indicating that membrane-bound BAFF, unlike soluble BAFF, does not support naïve B cells (32). But when follicular B cells were restored by administration of recombinant soluble BAFF 3-mer, full maturation of B cells, measured by CD23 expression, required membrane-bound BAFF (32). In mice with non-cleavable BAFF, membrane-bound BAFF co-localized with medullary fibroblastic reticular cells in lymph nodes. These cells make more direct contacts with plasma cells than any other cell type, and they can support survival of plasma cells *in vitro*, alone or together with macrophages, while other lymph node cell types cannot (33). This suggests that membrane-bound BAFF may be more important for plasma cells than for naïve B cells, possibly through activation of the receptors TACI and BCMA, and that inhibition of membrane-bound BAFF might be relevant to the mechanism of action of belimumab in SLE patients.

**Figure 6:**
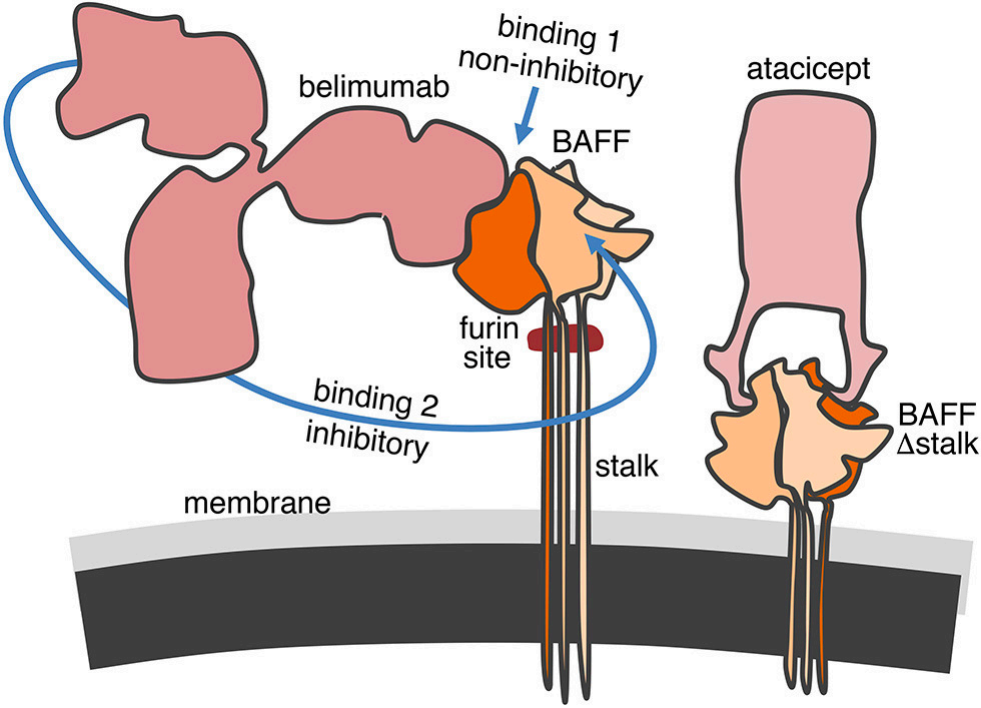
Hypothetical model for the binding of belimumab and atacicept to membrane-bound BAFF. The 66 amino acids-long stalk of BAFF contains the furin cleavage site and, if linear and extended, could have three times the height of the globular TNF homology domain. In contrast, the engineered stalk-less BAFF is much closer to the membrane. Atacicept binds BAFF from the side opposite to the membrane and has free access regardless of the length of the stalk. Belimumab is not only bulkier but also binds more on the side of BAFF along the entire height of the TNF homology domain. The first binding of belimumab to membrane-bound BAFF is probably always easy, but insufficient to inhibit the biological activity of BAFF. Inhibition only takes place upon binding of the second arm of the antibody to membrane-bound BAFF. Movements of the antibody to reach its second binding site might be compromised for steric hindrance reasons if BAFF is located too close to the cell membrane in BAFF Δstalk.

In this study, we have observed that belimumab binds normally to BAFF Δstalk overexpressed in CHO cells but was less efficient than atacicept to inhibit it. This was however not the case in furin-deficient U937 cells in which both atacicept and belimumab similarly inhibited full-length membrane-bound BAFF (and residual soluble BAFF). Thus, BAFF binding and BAFF inhibitory activities did not correlate well only for the pair of belimumab - BAFF Δstalk, but not for the pairs belimumab—full length BAFF, atacicept—BAFF Δstalk or atacicept—full-length BAFF. How can these results be reconciled in a molecular model? We have previously observed that atacicept binds and inhibits BAFF even when it is densely packed as 60-mers (30). It is therefore not surprising that atacicept can bind and inhibit membrane-bound BAFF irrespective of the length of the stalk region, as observed experimentally. We have also observed that, in contrast to intact belimumab, Fab fragments of belimumab did not or only very poorly inhibit the activity of BAFF 3-mers, probably because monovalent Fab can be displaced from BAFF by BAFFR (30). This would support a hypothetic model in which the first monovalent binding of belimumab to membrane-bound BAFF would allow cell staining by flow cytometry but would be poorly inhibitory. Membrane-bound BAFF would only be inhibited upon binding of the second arm of the antibody that increases binding avidity, preventing efficient displacement by BAFFR. The molecular “gymnastic” that belimumab must accomplish to reach its second binding site on membrane-bound BAFF might be partially impaired for steric hindrance reasons in the specific case of BAFF Δstalk that is positioned very close to the cell membrane (Figure 6).

Interestingly, we find that belimumab does not activate complement and Fc receptors *in vitro*. If this holds true *in vivo*, belimumab should not act by depleting cells expressing membrane-BAFF, in line with the current view of the mechanism of action of belimumab.

Belimumab was the first targeted treatment, approved in 2011, for the treatment of SLE, but its clinical efficacy is limited and not all patients benefit from treatment; this also holds true for other BAFF-blocking agents that have been tested in clinical trials (34). Loss of tolerance and development of auto-immunity is multifactorial, and SLE likely encompasses a spectrum of conditions with distinct etiologies that may be more or less dependent on BAFF (35, 36). This diversity is also reflected in the various spontaneous or induced mouse models of the disease (37). Different forms of BAFF and APRIL may trigger BAFFR, TACI, and BCMA in distinct manners with effects that could both promote (e.g., stimulation of auto-reactive B cells) or counteract (e.g., stimulation of regulatory B cells) development of symptoms. Indeed, in a mouse model of lupus, intricate positive or negative actions of the different receptors on disease severity have been described (38). In this context, it might not be superfluous to know the fine specificity of belimumab and other BAFF inhibitors.

## Data availability

The authors declare that the data supporting the finding of this study are available within the article, in its Supplementary Information File and as a dataset doi: 10.5281/zenodo.1481232.

## Author contributions

PS and MV conceived experiments. CK-Q, DC, LW, and PS performed experiments with help of MV and CJ. PS wrote the paper. All authors reviewed the results and approved the final version of the manuscript.

### Conflict of interest statement

PS receives research funds from Merck, KGaA for related research. The remaining authors declare that the research was conducted in the absence of any commercial or financial relationships that could be construed as a potential conflict of interest.

## Abbreviations

BAFF: B cell activating factor of the TNF family
APRIL: A proliferation-inducing ligand
BAFFR: BAFF receptor
TACI: Transmembrane activator and CAML interactor
BCMA: B cell maturation antigen
SLE: systemic lupus erythematosus.

## References

[1] Bossen C Schneider P. BAFF, APRIL and their receptors: structure, function and signaling. Semin Immunol. (2006) 18:263–75. 10.1016/j.smim.2006.04.006 16914324

[2] Gross JA Dillon SR Mudri S Johnston J Littau A Roque R. TACI-Ig neutralizes molecules critical for B cell development and autoimmune disease impaired B cell maturation in mice lacking BLyS. Immunity (2001) 15:289–302. 10.1016/S1074-7613(01)00183-2 11520463

[3] Schiemann B Gommerman JL Vora K Cachero TG Shulga-Morskaya S Dobles M. An essential role for BAFF in the normal development of B cells through a BCMA-independent pathway. Science (2001) 293:2111–4. 10.1126/science.1061964 11509691

[4] Sasaki Y Casola S Kutok JL Rajewsky K Schmidt-Supprian M. TNF family member B cell-activating factor (BAFF) receptor-dependent and -independent roles for BAFF in B cell physiology. J Immunol. (2004) 173:2245–52. 10.4049/jimmunol.173.4.2245 15294936

[5] Shulga-Morskaya S Dobles M Walsh ME Ng LG MacKay F Rao SP. B cell-activating factor belonging to the TNF family acts through separate receptors to support B cell survival and T cell-independent antibody formation. J Immunol. (2004) 173:2331–41. 10.4049/jimmunol.173.4.2331 15294946

[6] Benson MJ Dillon SR Castigli E Geha RS Xu S Lam KP. Cutting edge: the dependence of plasma cells and independence of memory B cells on BAFF and APRIL. J Immunol. (2008) 180:3655–9. 10.4049/jimmunol.180.6.3655 18322170

[7] Moore PA Belvedere O Orr A Pieri K LaFleur DW Feng P. BLyS: member of the tumor necrosis factor family and B lymphocyte stimulator. Science (1999) 285:260–3. 10.1126/science.285.5425.260 10398604

[8] Schneider P MacKay F Steiner V Hofmann K Bodmer JL Holler N. BAFF, a novel ligand of the tumor necrosis factor family, stimulates B cell growth. J Exp Med. (1999) 189:1747–56. 10.1084/jem.189.11.1747 10359578 PMC2193079

[9] Turpeinen H Ortutay Z Pesu M. Genetics of the first seven proprotein convertase enzymes in health and disease. Curr Genomics (2013) 14:453–67. 10.2174/1389202911314050010 24396277 PMC3867721

[10] Stohl W. Therapeutic targeting of the BAFF/APRIL axis in systemic lupus erythematosus. Expert Opin Ther Targets (2014) 18:473–89. 10.1517/14728222.2014.888415 24521424

[11] Samy E Wax S Huard B Hess H Schneider P. Targeting BAFF and APRIL in systemic lupus erythematosus and other antibody-associated diseases. Int Rev Immunol. (2017) 36:3–19. 10.1080/08830185.2016.1276903 28215100

[12] Furie R Petri M Zamani O Cervera R Wallace DJ Tegzova D. A phase III, randomized, placebo-controlled study of belimumab, a monoclonal antibody that inhibits B lymphocyte stimulator, in patients with systemic lupus erythematosus. Arthritis Rheum. (2011) 63:3918–30. 10.1002/art.30613 22127708 PMC5007058

[13] Navarra SV Guzman RM Gallacher AE Hall S Levy RA Jimenez RE. Efficacy and safety of belimumab in patients with active systemic lupus erythematosus: a randomised, placebo-controlled, phase 3 trial. Lancet (2011) 377:721–31. 10.1016/S0140-6736(10)61354-2 21296403

[14] Baker KP Edwards BM Main SH Choi GH Wager RE Halpern WG. Generation and characterization of LymphoStat-B, a human monoclonal antibody that antagonizes the bioactivities of B lymphocyte stimulator. Arthritis Rheum. (2003) 48:3253–65. 10.1002/art.11299 14613291

[15] FDA (2017). “Benlysta (belimumab).” Benlysta Package Insert.

[16] Schuepbach-Mallepell S Das D Willen L Vigolo M Tardivel A Lebon L. Stoichiometry of heteromeric BAFF and APRIL cytokines dictates their receptor-binding and signaling propoerties. J Biol Chem. (2015) 290:16330–42. 10.1074/jbc.M115.661405 25953898 PMC4481231

[17] Merrill JT Wallace DJ Wax S Kao A Fraser PA Chang P. Efficacy and safety of atacicept in patients with systemic Lupus Erythematosus: results of a twenty-four-week, multicenter, randomized, double-blind, placebo-controlled, parallel-Arm, Phase IIb study. Arthritis Rheumatol. (2018) 70:266–76. 10.1002/art.40360 29073347 PMC6099253

[18] Nardelli B Belvedere O Roschke V Moore PA Olsen HS Migone TS. Synthesis and release of B-lymphocyte stimulator from myeloid cells. Blood (2001) 97:198–204. 10.1182/blood.V97.1.198 11133761

[19] Schneider P. Production of recombinant TRAIL and TRAIL receptor:Fc chimeric proteins. Meth Enzymol. (2000) 322:322–45. 10.1016/S0076-6879(00)22031-4 10914028

[20] Holler N Kataoka T Bodmer JL Romero P Romero J Deperthes D. Development of improved soluble inhibitors of FasL and CD40L based on oligomerized receptors. J Immunol Methods (2000) 237:159–73. 10.1016/S0022-1759(99)00239-2 10725460

[21] Tom R Bisson L Durocher Y Transfection of HEK293-EBNA1 cells in suspension with linear PEI for production of recombinant proteins. CSH Protoc. 3:1–4. (2008). 10.1101/pdb.prot4977 21356793

[22] Shalem O Sanjana NE Hartenian E Shi X Scott DA Mikkelson T. Genome-scale CRISPR-Cas9 knockout screening in human cells. Science (2014) 343:84–7. 10.1126/science.1247005 24336571 PMC4089965

[23] Nys J Smulski CR Tardivel A Willen L Kowalczyk C Donze O. No evidence that soluble TACI induces signalling via membrane-expressed BAFF and APRIL in myeloid cells. PLoS ONE (2013) 8:e61350. 10.1371/journal.pone.0061350 23620746 PMC3631189

[24] Schneider P Willen L Smulski CR. Tools and techniques to study ligand-receptor interactions and receptor activation by TNF superfamily members. Methods Enzymol. (2014) 545:103–25. 10.1016/B978-0-12-801430-1.00005-6 25065888

[25] Cachero TG Schwartz IM Qian F Day E. S. Bossen C Ingold K. Formation of virus-like clusters is an intrinsic property of the tumor necrosis factor family member BAFF (B cell activating factor). Biochemistry (2006) 45:2006–13. 10.1021/bi051685o 16475789

[26] Yan L Hu R Tu S Cheng WJ Zheng Q Wang JW. Establishment of a cell model for screening antibody drugs against rheumatoid arthritis with ADCC and CDC. Int J Clin Exp Med. (2015) 8:20065–71. 26884918 PMC4723763

[27] Liu Y Xu L Opalka N Kappler J Shu HB Zhang G. Crystal structure of sTALL-1 reveals a virus-like assembly of TNF family ligands. Cell (2002) 108:383–94. 10.1016/S0092-8674(02)00631-1 11853672

[28] Nicoletti AM Kenny CH Khalil AM Pan Q Ralph KL Ritchie J. Unexpected potency differences between B-Cell-Activating Factor (BAFF) antagonist antibodies against various forms of BAFF: Trimer, 60-Mer, and membrane-bound. J Pharmacol Exp Ther. (2016) 359:37–44. 10.1124/jpet.116.236075 27440419

[29] Shin W Lee HT Lim H Lee SH Son JY Lee JU. BAFF-neutralizing interaction of belimumab related to its therapeutic efficacy for treating systemic lupus erythematosus. Nat Commun. (2018) 9:1200. 10.1038/s41467-018-03620-2 29572471 PMC5865148

[30] Vigolo M Chambers MG Willen L Chevalley D Maskos K Lammens A. A loop region of BAFF controls B cell survival and regulates recognition by different inhibitors. Nat Commun. (2018) 9:1199. 10.1038/s41467-018-03323-8 29572442 PMC5865128

[31] Halpern WG Lappin P Zanardi T Cai W Corcoran M Zhong J. Chronic administration of belimumab, a BLyS antagonist, decreases tissue and peripheral blood B-lymphocyte populations in cynomolgus monkeys: pharmacokinetic, pharmacodynamic, and toxicologic effects. Toxicol Sci. (2006) 91:586–99. 10.1093/toxsci/kfj148 16517838

[32] Bossen C Tardivel A Willen L Fletcher CA Perroud M Beermann F. Mutation of the BAFF Furin cleavage site impairs B cell homeostasis and anitibody responses. Eur J Immunol. (2011) 41:787–97. 10.1002/eji.201040591 21287546

[33] Huang HY Rivas-Caicedo A Renevey F Cannelle H Peranzoni E Scarpellino L. Identification of a new subset of lymph node stromal cells involved in regulating plasma cell homeostasis. Proc Natl Acad Sci USA. (2018) 115:E6826–E6835. 10.1073/pnas.1712628115 29967180 PMC6055158

[34] Stohl W. Inhibition of B cell activating factor (BAFF) in the management of systemic lupus erythematosus (SLE). Expert Rev Clin Immunol. (2017) 13:623–33. 10.1080/1744666X.2017.1291343 28164726

[35] Vincent FB Morand EF Schneider P Mackay F. The BAFF/APRIL system in SLE pathogenesis. Nat Rev Rheumatol. (2014) 10:365–73. 10.1038/nrrheum.2014.33 24614588

[36] Tsokos GC Lo MS Costa Reis P Sullivan KE. New insights into the immunopathogenesis of systemic lupus erythematosus. Nat Rev Rheumatol. (2016) 12:716–30. 10.1038/nrrheum.2016.186 27872476

[37] Li W Titov AA Morel L. An update on lupus animal models. Curr Opin Rheumatol. (2017) 29:434–41. 10.1097/BOR.0000000000000412 28537986 PMC5815391

[38] Jacob CO Yu N Sindhava V Cancro MP Pawar RD Putterman C. Differential development of systemic lupus erythematosus in NZM 2328 mice deficient in discrete pairs of BAFF receptors. Arthritis Rheumatol (2015) 67:2523–35. 10.1002/art.39210 25989238

