# Supplementary material for: Inhibition of Membrane-Bound BAFF by the Anti-BAFF Antibody Belimumab
Source: Front Immunol. 2018 Nov 20;9:2698. doi: 10.3389/fimmu.2018.02698 (PMC6256835; doi:10.3389/fimmu.2018.02698)
Supplement: Supplementary file 1 [file Table_1.PDF]

SUPPLEMENTARY INFORMATION

Inhibition of membrane-bound BAFF by the anti-BAFF antibody belimumab.

Christine Kowlaczyk-Quintas, Dehlia Chevalley, Laure Willen, Michele Vigolo and Pascal Schneider

Supplementary Table 1. Plasmids used in this study.

Plasmid	Designation	Protein encoded	Vector
ps515	EGFP	EGFP full (aa 1-239)	pcDNA3.1 zeo
ps544	hBAFF	hBAFF (aa 1-285)	PCR3
ps739	hBCMA-Fc	Ig signal-EVKLVPRGS-hBCMA (aa 2-54)-VD-hIgG1 (aa 245-470)	PCR3
ps1196	Fc-PreSci-hBAFF	HA signal LD h Fc (h IgG1 aa245-470) RSPQPQPKPQPKPEPEGSLEVLFQGPGSL h BAFF (aa136-285)	PCR3
ps1282	hBCMA-COMP-Flag	Ig signal-EVKLVPRGS-hBCMA (aa 2-54)-VD-PQPQPKPQPKPEPELEGGCC-hCOMP (aa 32-80)-EF-Flag	PCR3
ps1377	pMSCS-puro	Modified pMSCV-puro (Clontech) with HindIII-BglII-EcoRI-NotI-XhoI-HpaI-ApaI sites	ps1377
ps2308	hBAFFR:Fas	HA signal-LE-hBAFFR (aa 2-71)-EFGSVD-hFas (aa 169-355)	ps1377
ps2809	hBAFF-uncleavable 1	hBAFF (aa 1-132; Q84H)-L-hBAFF (aa 136-285)	PCR3
ps2825	Fc-hBAFF	HA signal LD h Fc (h IgG1 aa245-470) RSPQPQPKPQPKPEPEGSL h BAFF (aa136-285)	PCR3
ps3296	hBAFF-uncleavable 2	hBAFF (aa 1-285, Δ aa 85-136)	PCR3
ps3344	psPAX2	Agggene #12260	psPAX2
ps3345	VSV-G	Addgene #8454	PCMV
ps3391	Lenticrispr v2	Addgene #52961	ps3391
ps3511	gRNA hFurin for CRISP/Cas9	5'CACCG**AAGTGCACGGAGTCTCACAC**-3' and 5'-AAAC**TGGTGAGACTCCGTGCACTT**G-3' annealed and cloned into BsmB1 sites of ps3391	ps3391
ps3515	gRNA hBAFF for CRISP/Cas9	5'CACCG**ACTGATAAGACCTACGCCAT**-3' and 5'-AAAC**ATGGCGTAGGTCTTATCAGT**C-3' annealed and cloned into BsmB1 sites of ps3391	ps3391

Flag = DYKDDDDK HA signal=MAIIYLILLFTAVRG Ig signal=MNFGFSLIFLVLVLKG
