# Supplementary material for: Inhibition of Membrane-Bound BAFF by the Anti-BAFF Antibody Belimumab
Source: Front Immunol. 2018 Nov 20;9:2698. doi: 10.3389/fimmu.2018.02698 (PMC6256835; doi:10.3389/fimmu.2018.02698)
Supplement: Supplementary file 2 [file Data_Sheet_1.pdf]

Quintas et al | Figure 1 | Panel A

Cells:	293T-544 (BAFF full) + EGFP-515, transient			293T-2809 (BAFF ΔFS) + EGFP-515, transient			293T-3296 (BAFF Δstalk) + EGFP-515, transient			293T-015 (Mock) + EGFP-515, transient		
Staining reagent:	atacicept	atacicept	atacicept	atacicept	atacicept	atacicept	atacicept	atacicept	atacicept	atacicept	atacicept	atacicept
Measure: [ng/ml]	MFI of atacicept staining (FL2) for cells having 2x10e4 to 2x10e5 of EGFP (FL1)			MFI of atacicept staining (FL2) for cells having 2x10e4 to 2x10e5 of EGFP (FL1)			MFI of atacicept staining (FL2) for cells having 2x10e4 to 2x10e5 of EGFP (FL1)			MFI of atacicept staining (FL2) for cells having 2x10e4 to 2x10e5 of EGFP (FL1)		
100	190259	170898	195676	560693	698693	705693	43024	45573	46044	1716	1864	1846
10	145194	154782	149990	586693	686693	684693	40923	40495	42505	1751	2050	1779
1	26170	27268	25940	120148	111893	108032	20357	23227	22972	1839	1965	1849
0.1	4785	4918	5276	16381	15774	15949	4710	5256	5061	1721	1955	1891
Ø	1888	1956	2087	1591	1657	2683	1686	1938	2306	1710	2072	2148

Cells:	293T-544 (BAFF full) + EGFP-515, transient			293T-2809 (BAFF ΔFS) + EGFP-515, transient			293T-3296 (BAFF Δstalk) + EGFP-515, transient			293T-015 (Mock) + EGFP-515, transient		
Staining reagent:	belimumab	belimumab	belimumab	belimumab	belimumab	belimumab	belimumab	belimumab	belimumab	belimumab	belimumab	
[ng/ml]	MFI of atacicept staining (FL2) for cells having 2x10e4 to 2x10e5 of EGFP (FL1)			MFI of atacicept staining (FL2) for cells having 2x10e4 to 2x10e5 of EGFP (FL1)			MFI of atacicept staining (FL2) for cells having 2x10e4 to 2x10e5 of EGFP (FL1)			MFI of atacicept staining (FL2) for cells having 2x10e4 to 2x10e5 of EGFP (FL1)		
100	353592	342736	367421	1346383	1376383	1176383	108049	108490	118207	1978	2127	nd
10	417140	371632	388386	1136383	1106383	1006383	81897	79935	82305	1885	2039	nd
1	75182	80876	78225	186334	227418	157711	18334	18923	19247	1925	1930	nd
0.1	6445	7817	7200	26887	35014	18950	5874	4053	3951	1840	1997	nd
Ø	2696	*11166**	1321	3037	1882	1108	1803	2058	2165	1924	2020	nd

Cells:	293T-544 (BAFF full) + EGFP-515, transient			293T-2809 (BAFF ΔFS) + EGFP-515, transient			293T-3296 (BAFF Δstalk) + EGFP-515, transient			293T-015 (Mock) + EGFP-515, transient		
Staining reagent:	denosumab	denosumab	denosumab	denosumab	denosumab	denosumab	denosumab	denosumab	denosumab	denosumab	denosumab	denosumab
[ng/ml]	MFI of atacicept staining (FL2) for cells having 2x10e4 to 2x10e5 of EGFP (FL1)			MFI of atacicept staining (FL2) for cells having 2x10e4 to 2x10e5 of EGFP (FL1)			MFI of atacicept staining (FL2) for cells having 2x10e4 to 2x10e5 of EGFP (FL1)			MFI of atacicept staining (FL2) for cells having 2x10e4 to 2x10e5 of EGFP (FL1)		
100	2429	2138	2425	1559	1050	*-510**	2243	2267	2887	2238	1859	2151
10	3009	2161	2447	1907	2590	1076	3059	2223	2264	2089	1956	2004
1	2191	*30844**	2335	1801	2169	1267	2214	2531	2145	2105	2079	2241
0.1	2241	*13200**	2225	2995	2864	2690	2817	2376	2202	2126	2412	2179
Ø	2643	*6909**	1951	2765	1792	2333	2368	2344	2179	2164	1999	2727

Remarks: Values in blue were excluded from the analysis
Background was substracted to get similar MFI in unstained cells
Background substracted from 293T-015 Atacicept: 171
Background substracted from 293T-015 Belimumab: 192
Background substracted from 293T-015 Denosumab: 170

Background substracted from 293T-544 Atacicept: 1037
Background substracted from 293T-544 Belimumab: 3860
Background substracted from 293T-544 Denosumab: 1063

Background substracted from 293T-2809 Atacicept: 3307
Background substracted from 293T-2809 Belimumab: 3613
Background substracted from 293T-2809 Denosumab: 3170

Background substracted from 293T-3296 Atacicept: 0
Background substracted from 293T-3296 Belimumab: 0
Background substracted from 293T-3296 Denosumab: 0

Quintas et al Figure 1 Panel B

Cells:	293T-544 (BAFF full) + EGFP-515, transient		
Staining reagent:	BCMA-COMP-Flag-1282		
Measure:	MFI of atacicept staining (FL2) for cells having 2x10e4 to 2x10e5 of EGFP (FL1)		
Competitor:			
None:	25321	25141	26095
Atacicept:	1182	1232	991
Belimumab:	1627	1675	1789
Denosumab	25249	25650	24517

Cells:	293T-2809 (BAFF ΔFS) + EGFP-515, transient		
Staining reagent:	BCMA-COMP-Flag-1282		
Measure:	MFI of atacicept staining (FL2) for cells having 2x10e4 to 2x10e5 of EGFP (FL1)		
Competitor:			
None:	100458	104485	107111
Atacicept:	2047	1859	2013
Belimumab:	3918	4531	3384
Denosumab	109682	105439	106517

Cells:	293T-3296 (BAFF Δstalk) + EGFP-515, transient		
Staining reagent:	BCMA-COMP-Flag-1282		
Measure:	MFI of atacicept staining (FL2) for cells having 2x10e4 to 2x10e5 of EGFP (FL1)		
Competitor:			
None:	36885	37654	37941
Atacicept:	1583	1296	1688
Belimumab:	8504	7809	7353
Denosumab	36585	36623	36576

Cells:	293T-015 (Mock) + EGFP-515, transient		
Staining reagent:	BCMA-COMP-Flag-1282		
Measure:	MFI of atacicept staining (FL2) for cells having 2x10e4 to 2x10e5 of EGFP (FL1)		
Competitor:			
None:	2169	1811	1743
Atacicept:	1279	1442	1314
Belimumab:	1769	1736	1614
Denosumab	1805	1767	1537

Quintas et al Figure 2 Panel A

Cells:	CHO-3296 clone 7	CHO-3296 clone 7	CHO-3296 clone 7
Staining	Atacicept	Belimumab	Denosumab
Measure:	MFI	MFI	MFI
ng/staining			
111.1111	4612	5608	1237
37.03704	4218	4937	1233
12.34568	3652	4191	1263
4.115226	3097	3193	1238
1.371742	2705	2424	1262
0.3429355	1285	1243	1294

Quintas et al Figure 2 Panel B

	Target cells:	Jurkat JOM2 BAFFR:Fas-2308 cl21			Jurkat JOM2 BAFFR:Fas-2308 cl21			Jurkat JOM2 BAFFR:Fas-2308 cl21		
	Effector:	Conditioned medium of CHO-3296			Conditioned medium of CHO			Fc-hBAFF-1196		
	Measure:	Reporter cell viability [A492]			Reporter cell viability [A492]			Reporter cell viability [A492]		
Fc-BAFF-1196 ng/ml	Conditioned medium µl									
50	50	0.857	0.78	0.734	0.538	0.668	0.588	0.191	0.196	0.212
25	25	0.996	0.85	0.84	0.718	0.775	0.684	0.193	0.2	0.201
12.5	12.5	0.942	0.91	0.862	0.74	0.801	0.76	0.204	0.196	0.207
6.25	6.25	0.916	0.844	0.914	0.809	0.797	0.759	0.2	0.198	0.202
3.125	3.125	0.904	0.98	0.907	0.839	0.814	0.791	0.202	0.194	0.207
1.5625	1.5625	0.92	1.006	0.94	0.869	0.821	0.85	0.204	0.209	0.204
0.78125	0.78125	0.907	1.071	0.979	0.895	0.796	0.914	0.207	0.206	0.213
0.390625	0.390625	0.901	1.076	0.964	0.903	0.762	0.841	0.214	0.221	0.209
0.1953125	0.1953125	0.88	1.045	0.86	0.864	0.889	0.886	0.201	0.199	0.224
0.09765625	0.09765625	0.88	0.975	0.938	0.83	0.811	0.792	0.2	0.22	0.227
0.04882812	0.04882812	1.01	0.972	0.995	0.987	0.878	0.832	0.202	0.224	0.245
0.02441406	0.02441406	0.977	1.063	0.885	0.971	0.858	0.851	0.22	0.269	0.331
0.01220703	0.012207	0.927	0.934	0.877	0.889	0.895	0.845	0.222	0.363	0.502
0.006103516	0.0061035	0.889	0.933	0.887	0.901	0.853	0.85	0.279	0.487	0.741
0.003051758	0.003051758	0.951	0.953	0.893	0.918	0.848	0.816	0.393	0.659	0.678
Ø	Ø	0.997	0.982	0.867	0.905	0.842	0.855	0.822	0.808	0.83

Quintas et al Figure 2 Panel C

Effector cells:	CHO-3296 clone 7		CHO-3296 clone 7		CHO-3296 clone 7	
Target cells:	Jurkat JOM2 BAFFR:Fas-2308 cl21 (CFSE)		Jurkat JOM2 BAFFR:Fas-2308 cl21 (CFSE)		Jurkat JOM2 BAFFR:Fas-2308 cl21 (CFSE)	
Inhibitor:	Atacicept		Belimumab		Denosumab	
Measure:	% viability	% viability	% viability	% viability	% viability	% viability
Inhibitor [µg/ml]						
20	82.5047	81.3423	76.63818	72.79793	20.35365	16.3959
6.666667	87.89124	82.3476	73.16479	63.36178	19.51693	14.46265
2.222222	86.90115	83.25733	57.38533	45.15926	20.31434	13.51846
0.7407407	*50.98103**	77.32308	33.68484	36.10007	23.10276	14.93348
0.2469136	75.4828	67.26884	25.74807	28.41499	22.41168	17.02424
0.08230453	39.54807	38.572	24.1997	22.57478	21.0996	13.27375
0.02743484	28.05652	22.5418	26.21011	16.25483	17.97588	16.16508
0.005486968	14.81746	19.00875	20.39606	17.7794	21.46609	13.7893

Remark: Value in blue was excluded from the analysis

Quintas et al	Figure 2	Panel D

Effector :	Fc-BAFF-1196 (100 ng/ml)	Fc-BAFF-1196 (100 ng/ml)	Fc-BAFF-1196 (100 ng/ml)
Target cells:	Jurkat JOM2 BAFFR:Fas-2308 cl21 (CFSE)	Jurkat JOM2 BAFFR:Fas-2308 cl21 (CFSE)	Jurkat JOM2 BAFFR:Fas-2308 cl21 (CFSE)
Inhibitor:	Atacicept	Belimumab	Denosumab
Measure:	% viability	% viability	% viability
Inhibitor [µg/ml]			
2.222222	61.54573	66.17574	7.759914
0.7407407	71.87395	74.48164	10.7481
0.2469136	71.02573	64.16835	12.4952
0.08230453	70.13562	67.15076	12.28478
0.02743484	21.89094	35.14849	9.199497
0.005486968	8.719499	10.76799	10.8003

Quintas et al	Figure 2	Panel E

Effector cells:	CHO	CHO	CHO
Target cells:	Jurkat JOM2 BAFFR:Fas-2308 cl21 (CFSE)	Jurkat JOM2 BAFFR:Fas-2308 cl21 (CFSE)	Jurkat JOM2 BAFFR:Fas-2308 cl21 (CFSE)
Inhibitor:	Atacicept	Belimumab	Denosumab
Measure:	% viability	% viability	% viability
Inhibitor [µg/ml]			
20	90.09245	90.85391	86.54024
6.666667	87.90071	82.55782	84.3914
2.222222	90.13409	87.45382	85.46166
0.7407407	88.00522	87.08832	86.58247
0.2469136	87.75202	86.94717	84.78233
0.08230453	88.5471	86.57661	84.72982
0.02743484	89.07141	88.69598	86.08019
0.005486968	87.75867	81.68044	85.54778

Quintas et al	Figure 2	Panel F

Effectors:	CHO + Fc-BAFF-1196 (100 ng/ml)	CHO + Fc-BAFF-1196 (100 ng/ml)	CHO + Fc-BAFF-1196 (100 ng/ml)
Target cells:	Jurkat JOM2 BAFFR:Fas-2308 cl21 (CFSE)	Jurkat JOM2 BAFFR:Fas-2308 cl21 (CFSE)	Jurkat JOM2 BAFFR:Fas-2308 cl21 (CFSE)
Inhibitor:	Atacicept	Belimumab	Denosumab
Measure:	% viability	% viability	% viability
Inhibitor [µg/ml]			
2.222222	76.96738	80.61203	18.87001
0.7407407	*45.50512**	82.35783	23.98705
0.2469136	71.29617	75.66724	19.36922
0.08230453	81.58752	77.05611	16.0623
0.02743484	62.77562	70.06744	9.78158
0.005486968	25.10397	17.97143	15.30033

Remark: Value in blue was excluded from the analysis

Quintas et al Figure 3 Panel A

Cells:	U937 BAFF-ko	U937 WT	U937 Furin-ko
Staining	Biotinylated atacicept	Biotinylated atacicept	Biotinylated atacicept
Measure:	MFI	MFI	MFI
ng/staining			
2000	1098	1173	8393
400	995	1110	12508
80	915	1008	10917
16	899	947	8422
3.2	917	900	4060
0.64	1021	934	855

Quintas et al Figure 3 Panel B

Cells:	U937 BAFF-ko	U937 WT	U937 Furin-ko
Staining	Biotinylated belimumab	Biotinylated belimumab	Biotinylated belimumab
Measure:	MFI	MFI	MFI
ng/staining			
2000	835	835	6120
400	898	905	6196
80	898	902	6042
16	867	868	4186
3.2	848	876	1983
0.64	861	853	802

Quintas et al Figure 3 C

h BAFF ELISA

Standard pg/ml	Standard Log (pg/ml)	Standard A450	log (A450)
1000	3	3.19	0.503790683
500	2.69897	1.965	0.293362555
250	2.39794	1.206	0.081347308
125	2.09691	0.775	-0.1106983
62.5	1.79588	0.483	-0.31605287
31.25	1.49485	0.31	-0.50863831
15.625	1.19382	0.232	-0.63451202
0	#NUM!	0.215	-0.66756154

Regression curve

Slope	0.6722
X-intercept	-1.52

Formula to convert A450 to ng/ml of hBAFF: =POWER(10,(([log A450]+1.52)/0.6722))*[dilution factor]/1000

Valid for 0.22 < A450 < 3.2

S/N of cells:	U937 BAFF-ko-3515		U937 Furin-ko-3511		U937 WT		U937 WT		U937 WT	
Dilution factor in ELISA:	2	2	2	2	4	4	40	40	400	400
	A450	A450	A450	A450	A450	A450	A450	A450	A450	A450
Time [h]	replicate 1	replicate 2	replicate 1	replicate 2	replicate 1	replicate 2	replicate 1	replicate 2	replicate 1	replicate 2
1	0.176	0.177	0.205	0.232	0.789	1.427	0.24	0.257	0.194	0.205
6	0.174	0.16	0.26	0.24	3.289	3.511	0.548	0.575	0.215	0.197
22	0.155	0.168	0.505	0.522	4	4	1.631	1.633	0.335	0.328
72	0.181	0.168	1.435	1.468	4	4	3.855	3.672	0.817	0.817

excluded from analysis

	log (A450)	log (A450)	log (A450)	log (A450)	log (A450)	log (A450)	log (A450)	log (A450)	log (A450)	log (A450)
Time [h]	replicate 1	replicate 2	replicate 1	replicate 2	replicate 1	replicate 2	replicate 1	replicate 2	replicate 1	replicate 2
1	-0.754487332	-0.75202673	-0.688246139	-0.634512015	-0.102922997	0.154423973	-0.619788758	-0.590066877	-0.71219827	-0.688246139
6	-0.759450752	-0.79588002	-0.585026652	-0.619788758	0.517063873	0.545430829	-0.261219442	-0.240332155	-0.66756154	-0.705533774
22	-0.809668302	-0.77469072	-0.296708622	-0.282329497	0.602059991	0.602059991	0.212453961	0.212986185	-0.474955193	-0.484126156
72	-0.742321425	-0.77469072	0.156851901	0.166726056	0.602059991	0.602059991	0.586024382	0.564902673	-0.087777943	-0.087777943

hBAFF in undiluted S/N:	hBAFF [ng/ml]	hBAFF [ng/ml]	hBAFF [ng/ml]	hBAFF [ng/ml]	hBAFF [ng/ml]	hBAFF [ng/ml]	hBAFF [ng/ml]	hBAFF [ng/ml]	hBAFF [ng/ml]	hBAFF [ng/ml]
Time [h]	replicate 1	replicate 2	replicate 1	replicate 2	replicate 1	replicate 2	replicate 1	replicate 2	replicate 1	replicate 2
1	0.027532574	0.027765617	0.034545437	0.041526793	0.513071765	1.238856417	0.873497128	0.967113204	6.364851006	6.909087441
6	0.027068424	0.023892901	0.04919777	0.043674856	4.29045275	4.72828119	2.983238784	3.204505635	7.416383585	6.511824631
22	0.02279065	0.025691603	0.132087723	0.138756582	5.740474729	5.740474729	15.11295644	15.14053406	14.34575781	13.90209617
72	0.028704201	0.025691603	0.624601312	0.646088785	5.740474729	5.740474729	54.336592	50.54411909	54.03917273	54.03917273

	Log(hBAFF ng/ml)		Log(hBAFF ng/ml)		Log(hBAFF ng/ml)		Log(hBAFF ng/ml)		Log(hBAFF ng/ml)	
Log(time [h])	replicate 1	replicate 2	replicate 1	replicate 2	replicate 1	replicate 2	replicate 1	replicate 2	replicate 1	replicate 2
0	-1.560153182	-1.55649267	-1.461609307	-1.381671604	-0.289821884	0.093020975	-0.058738518	-0.014522687	0.803788242	0.839420689
0.7781513	-1.567537026	-1.62173111	-1.30805458	-1.359768514	0.632503124	0.674703296	0.474688016	0.50576104	0.870192184	0.813702696
1.3424227	-1.642243289	-1.5902088	-0.879137547	-0.857746406	0.758947809	0.758947809	1.179349431	1.180141194	1.156723495	1.143080288
1.8573325	-1.54205454	-1.5902088	-0.204397108	-0.189707797	0.758947809	0.758947809	1.735092396	1.703670632	1.732708692	1.732708692

Values used in Fig. 3C

Quintas et al Figure 3 Panel D

Target cells:		Jurkat JOM2 BAFFR:Fas-2308 cl21				Jurkat JOM2 BAFFR:Fas-2308 cl21				Jurkat JOM2 BAFFR:Fas-2308 cl21				
Effector:		S/N of U937 BAFF-ko-3515				S/N of U937 WT				S/N of U937 Furin-ko-3511				
Measure:		Reporter cell viability [A492]				Reporter cell viability [A492]				Reporter cell viability [A492]				
		Time 1 h	Time 6 h	Time 22 h	Time 72 h	Time 1 h	Time 6 h	Time 22 h	Time 72 h	Time 1 h	Time 6 h	Time 22 h	Time 72 h	
conditioned medium [µl]	log (S/N µl)													
50	1.69897	0.488	0.5	0.555	0.493	0.303	0.262	0.228	0.224	0.529	0.516	0.39	0.285	
25	1.39794001	0.53	0.486	0.485	0.464	0.35	0.269	0.239	0.231	0.492	0.516	0.444	0.304	
12.5	1.09691001	0.508	0.501	0.458	0.455	0.408	0.29	0.245	0.259	0.512	0.5	0.479	0.382	
6.25	0.79588002	0.487	0.502	0.471	0.455	0.436	0.314	0.25	0.261	0.523	0.505	0.512	0.388	
3.125	0.49485002	0.507	0.518	0.442	0.463	0.496	0.359	0.289	0.257	0.524	0.502	0.506	0.416	
1.5625	0.19382003	0.533	0.523	0.458	0.448	0.638	0.425	0.303	0.27	0.518	0.514	0.486	0.411	
0.78125	-0.10721	0.504	0.52	0.451	0.455	0.509	0.496	0.358	0.269	0.509	0.5	0.492	0.448	
0.390625	-0.40824	0.527	0.524	0.448	0.471	0.507	0.522	0.368	0.288	0.545	0.515	0.504	0.469	Excluded from the analysis
0.1953125	-0.70927	0.537	0.518	0.47	0.491	0.779	0.542	0.454	0.325	0.545	0.515	0.511	0.487	
0.09765625	-1.0103	0.526	0.517	0.453	0.505	0.553	0.554	0.503	0.365	0.603	0.509	0.519	0.449	
0.048828125	-1.31133	0.524	0.5	0.473	0.464	0.526	0.516	0.458	0.421	0.516	0.541	0.515	0.447	
0.024414063	-1.6123599	0.497	0.488	0.477	0.428	0.501	0.611	0.422	0.453	0.524	0.499	0.484	0.443	
0.012207031	-1.9133899	0.503	0.508	0.46	0.494	0.523	0.508	0.496	0.463	0.527	0.465	0.478	0.443	
0.006103516	-2.2144199	0.519	0.509	0.44	0.485	0.535	0.535	0.5	0.445	0.989	0.5	0.464	0.446	
0.003051758	-2.5154499	0.545	0.538	0.478	0.474	0.539	0.514	0.498	0.507	0.556	0.498	0.471	0.522	
0.001525879	-2.8164799	0.56	0.508	0.451	0.442	0.53	0.536	0.487	0.502	0.504	0.5	0.502	0.456	

		Cell viability [%], normalized				Cell viability [%], normalized				Cell viability [%], normalized				
conditioned medium [µl]	log (S/N µl)	Time 1 h	Time 6 h	Time 22 h	Time 72 h	Time 1 h	Time 6 h	Time 22 h	Time 72 h	Time 1 h	Time 6 h	Time 22 h	Time 72 h	
50	1.69897	84.4	94.0		108.5	18.8	4.4	2.9	-11.4	100.0	106.8	65.6	16.1	
25	1.39794001	99.3	88.7	111.8	95.5	35.5	7.0	6.9	-8.3	86.7	106.8	86.5	24.9	
12.5	1.09691001	91.5	94.4	100.4	91.5	56.0	14.7	9.1	3.9	93.9	100.4	100.0	60.8	Excluded for the determination of EC50
6.25	0.79588002	84.0	94.7	105.9	91.5	66.0	23.4	10.9	4.8	97.8	102.4	112.7	63.6	
3.125	0.49485002	91.1	100.8	93.7	95.1	87.2	39.9	25.1	3.1	98.2	101.2	110.4	76.5	
1.5625	0.19382003	100.4	102.6	100.4	88.4		64.1	30.2	8.7	96.1	106.0	102.7	74.2	
0.78125	-0.10721	90.1	101.5	97.5	91.5	91.8	90.1	50.2	8.3	92.8	100.4	105.0	91.2	
0.390625	-0.40824	98.2	103.0	96.2	98.7	91.1	99.6	53.8	16.6	105.7	106.4	109.7	100.9	
0.1953125	-0.70927	101.8	100.8	105.5	107.6		107.0	85.1	32.8	105.7	106.4	112.4	109.2	
0.09765625	-1.0103	97.9	100.4	98.3	113.8	107.4	111.4	102.9	50.2		104.0	115.4	91.7	
0.048828125	-1.31133	97.2	94.0	106.8	95.5	97.9	97.4	86.5	74.7	95.3	116.9	113.9	90.8	
0.024414063	-1.6123599	87.6	89.5	108.4	79.5	89.0		73.5	88.6	98.2	100.0	101.9	88.9	
0.012207031	-1.9133899	89.7	97.0	101.3	108.9	96.8	94.5	100.4	93.0	99.3	86.3	99.6	88.9	
0.006103516	-2.2144199	95.4	97.4	92.8	104.9	101.1	104.4	101.8	85.2		100.4	94.2	90.3	
0.003051758	-2.5154499	104.6	108.3	108.9	100.0	102.5	96.7	101.1	112.2	109.7	99.6	96.9	125.3	
0.001525879	-2.8164799	109.9	97.0	97.5	85.7	99.3	104.8	97.1	110.0	91.0	100.4	108.9	94.9	

Quintas et al Figure 4 Panel A

Effector :	None			None			None		
Target cells:	Jurkat JOM2 BAFFR:Fas-2308 cl21 (CFSE)			Jurkat JOM2 BAFFR:Fas-2308 cl21 (CFSE)			Jurkat JOM2 BAFFR:Fas-2308 cl21 (CFSE)		
Inhibitor:	Atacicept			Belimumab			Denosumab		
Measure:	% viability			% viability			% viability		
Inhibitor [ng/ml]									
20000	97.8	99.6	88	100.7	88.7	100.2	101.8	103.6	98
5000	93.8	98.4	93.3	101.1	98	96.2	97.8	96.4	96.7
1250	110.9	119.3	102.4	97.1	101.8	112.2	106.4	108.7	110.2
312.5	102.2	106.2	94	98	98.9	102.2	108.7	102.2	100.2
78.125	95.3	101.3	92.9	89.1	87.3	97.8	105.6	98.7	106
19.53125	113.3	110.9	112.7	96.7	84.4	91.3	104.2	103.6	107.3
4.882813	109.1	109.8	105.3	95.1	102.9	135.3	103.6	106	105.6
Ø	103.3	106	99.1	99.1	99.6	106.9	104.2	97.6	114.2

Quintas et al Figure 4 Panel B

Effector :	20 ng/ml of Fc-hBAFF-2825			20 ng/ml of Fc-hBAFF-2825			20 ng/ml of Fc-hBAFF-2825		
Target cells:	Jurkat JOM2 BAFFR:Fas-2308 cl21 (CFSE)			Jurkat JOM2 BAFFR:Fas-2308 cl21 (CFSE)			Jurkat JOM2 BAFFR:Fas-2308 cl21 (CFSE)		
Inhibitor:	Atacicept			Belimumab			Denosumab		
Measure:	% viability			% viability			% viability		
Inhibitor [ng/ml]									
20000	103.3	102.4	106.7	114.9	118.5	119.8	6.7	6.4	6.4
5000	92	98.4	99.1	91.8	115.8	117.3	7.3	6.7	7.5
1250	92.9	99.8	99.3	117.8	116.4	113.1	5.8	6.4	6.2
312.5	85.6	100.4	95.5	108.4	125.3	121.3	5.8	6	6.5
78.125	105.1	93.1	107.5	109.5	100.5	112.4	6	4	6.4
19.53125	12.7	9.6	11.6	7.5	8.4	7.6	5.3	7.3	7.8
4.882813	6.9	6.4	6.4	6.7	6.5	5.6	5.8	6.5	6.5
Ø	7.5	5.3	6.4	7.3	7.3	5.8	6.5	6.5	7.5

Quintas et al Figure 4 Panel C

Effector :	U937 BAFF-ko			U937 BAFF-ko			U937 BAFF-ko		
Target cells:	Jurkat JOM2 BAFFR:Fas-2308 cl21 (CFSE)			Jurkat JOM2 BAFFR:Fas-2308 cl21 (CFSE)			Jurkat JOM2 BAFFR:Fas-2308 cl21 (CFSE)		
Inhibitor:	Atacicept			Belimumab			Denosumab		
Measure:	% viability			% viability			% viability		
Inhibitor [ng/ml]									
20000	88.2	96.6	116.8	106	114.2	111.4	107.2	80.4	106.6
5000	99	120.8	106.6	98	95	104	97.4	87.2	88.8
1250	93.2	101.8	111	94.8	100.4	108	107.2	98	99.6
312.5	98.8	104.6	106	111.6	109.8	113	109.2	94	91
78.125	109.2	97.6	83	87	125.6	106.6	104.6	105	94
19.53125	87.2	83.2	108.6	108.8	105.6	108.6	107.4	108	104
4.882813	72.6*	96.8	69.8*	100.4	106.8	103.8	102	101.6	106.6
Ø	59.6*	90.6	95	97.2	99.8	96.2	97.6	95.8	91

Quintas et al Figure 4 Panel D

Effector :	U937 Furin-ko			U937 Furin-ko			U937 Furin-ko		
Target cells:	Jurkat JOM2 BAFFR:Fas-2308 cl21 (CFSE)			Jurkat JOM2 BAFFR:Fas-2308 cl21 (CFSE)			Jurkat JOM2 BAFFR:Fas-2308 cl21 (CFSE)		
Inhibitor:	Atacicept			Belimumab			Denosumab		
Measure:	% viability			% viability			% viability		
Inhibitor [ng/ml]									
20000	99.3	90	113.5	96.7	103.5	111.6	23.5	26.4	26.7
5000	83.5	83.8	112	102.2	107.5	112	25.6	22.7	22.5
1250	100.7	86.5	112.9	99.1	107.8	108.7	22.7	25.6	22.4
312.5	106.2	87.6	107.1	96.4	105.8	108	25.1	25.3	22.7
78.125	114	105.3	115.6	96.2	103.1	106.9	38.7	21.8	22.2
19.53125	81.6	77.5	84.2	70.7	79.3	82	21.6	32.4	22.7
4.882813	58.9	56.9	58.5	34.9	62	62.5	26	24	19.3
1.220703	36.4	44.9	49.3	29.6	37.8	48.4	nd	nd	nd
0.2	26.9	27.1	26.4	16.9	24.4	26.7	26.7	27.6	27.5

Quintas et al Figure 4 Panel E

Target cells:	Jurkat JOM2 BAFFR:Fas-2308 cl21	Jurkat JOM2 BAFFR:Fas-2308 cl21	Jurkat JOM2 BAFFR:Fas-2308 cl21	Jurkat JOM2 BAFFR:Fas-2308 cl21	Jurkat JOM2 BAFFR:Fas-2308 cl21
Effector:	Conditioned medium of U937 BAFF-ko-3515	Conditioned medium of U937 WT	Conditioned medium of U937 Furin-ko-3511	20 ng/ml Fc-hBAFF-2825	None
Measure:	Reporter cell viability [A492]	Reporter cell viability [A492]	Reporter cell viability [A492]	Reporter cell viability [A492]	Reporter cell viability [A492]
triplicates	1.187	0.292	0.95	0.322	1.418
	1.262	0.306	0.823	0.311	1.344
	1.307	0.306	0.944	0.312	1.322

Quintas et al Figure 5 Panel A

Reporter cells:	CHO-3296 clone 7		CHO-3296 clone 7		CHO-3296 clone 7		CHO-3296 clone 7		CHO-3296 clone 7		CHO-3296 clone 7	
Mediator	atacicept		belimumab		denosumab		adalimumab		BCMA-Fc		BCMA-Fc dimer	
Effector	normal human serum		normal human serum		normal human serum		normal human serum		normal human serum		normal human serum	
	cell viability [A492]		cell viability [A492]		cell viability [A492]		cell viability [A492]		cell viability [A492]		cell viability [A492]	
Effector [µg/ml]												
10	0.665	0.758	0.869	1.02	0.968	1.088	0.87	1.107	0.453	0.4	0.417	0.342
2	0.956	0.919	0.988	0.91	1.032	0.912	0.961	0.891	0.413	0.526	0.406	0.394
0.4	1.161	0.866	0.97	0.822	1.067	0.971	0.94	0.928	0.441	0.536	0.453	0.403
0.08	0.89	0.909	1.066	0.925	1.18	0.963	0.97	0.874	0.628	0.612	0.592	0.661
0.016	0.972	0.878	0.884	0.872	0.927	0.879	0.934	0.855	0.897	0.891	0.882	0.976
0.0032	0.893	0.873	1.043	0.797	0.855	0.886	0.827	0.835	0.861	0.921	0.889	0.789
0.00064	0.769	0.895	0.875	0.9	0.894	0.85	0.875	0.86	0.942	0.918	0.889	0.907
0.000064	0.72	0.862	0.982	0.91	1.004	0.881	0.902	0.807	0.855	0.934	0.898	0.715

Reporter cells:	CHO-3296 clone 7		CHO-3296 clone 7		CHO-3296 clone 7		CHO-3296 clone 7		CHO-3296 clone 7		CHO-3296 clone 7	
Mediator	atacicept		belimumab		denosumab		adalimumab		BCMA-Fc		BCMA-Fc dimer	
Effector	heat-inactivated serum		heat-inactivated serum		heat-inactivated serum		heat-inactivated serum		heat-inactivated serum		heat-inactivated serum	
	cell viability [A492]		cell viability [A492]		cell viability [A492]		cell viability [A492]		cell viability [A492]		cell viability [A492]	
Effector [µg/ml]												
10	0.799	0.702	0.85	0.796	0.944	0.687	0.889	0.901	0.792	0.787	0.659	0.93
2	0.827	0.88	0.805	0.818	0.828	0.985	0.856	0.84	0.834	0.939	0.879	1.028
0.4	0.863	0.885	0.85	0.87	0.891	0.981	0.934	0.806	0.855	0.906	0.843	0.964
0.08	0.958	0.897	0.904	0.955	0.943	0.979	0.962	0.798	0.996	0.896	0.966	0.806
0.016	0.976	0.946	0.975	0.881	0.945	0.881	0.899	0.788	0.915	0.92	0.928	0.901
0.0032	0.917	0.942	0.93	1.019	0.953	0.915	0.933	0.824	0.865	0.831	0.87	0.89
0.00064	0.877	0.853	0.843	0.897	0.894	0.85	0.85	0.784	0.9	0.8	0.871	0.871
0.000064	0.921	0.866	0.903	0.986	0.931	1.148	1.012	0.748	0.963	0.788	0.851	0.89

Reporter cells:	CHO cells		CHO cells		CHO cells		CHO cells		CHO cells		CHO cells	
Mediator	atacicept		belimumab		denosumab		adalimumab		BCMA-Fc		BCMA-Fc dimer	
Effector	normal human serum		normal human serum		normal human serum		normal human serum		normal human serum		normal human serum	
	cell viability [A492]		cell viability [A492]		cell viability [A492]		cell viability [A492]		cell viability [A492]		cell viability [A492]	
Effector [µg/ml]												
10	1.003	1.071	1.003	0.764	0.984	1.279	0.974	1.162	0.961	1.141	0.961	1.2
2	1.003	1.063	0.926	1.122	0.999	1.279	0.977	1.189	0.931	1.081	0.945	1.082
0.4	0.978	1.1	0.965	1.107	0.945	1.253	0.979	1.167	0.963	1.088	1.02	0.926
0.08	0.967	1.09	0.933	1.18	0.959	1.058	0.929	1.051	0.983	1.034	1.006	1.027
0.016	0.985	1.079	0.962	1.12	0.955	1.092	0.946	0.99	0.919	0.915	1.031	0.981
0.0032	0.944	1.088	0.943	1.138	0.922	1.064	0.968	0.999	1.006	1.023	1.07	1.021
0.00064	1.022	1.06	0.967	1.039	0.943	1.088	1.003	1.063	0.983	1.022	1	1.012
0.000064	0.988	0.98	0.994	0.809	0.982	1.048	1.016	1.025	1.066	1.004	1.028	1.057

Reporter cells:	CHO cells		CHO cells		CHO cells		CHO cells		CHO cells		CHO cells	
Mediator	atacicept		belimumab		denosumab		adalimumab		BCMA-Fc		BCMA-Fc dimer	
Effector	heat-inactivated serum		heat-inactivated serum		heat-inactivated serum		heat-inactivated serum		heat-inactivated serum		heat-inactivated serum	
	cell viability [A492]		cell viability [A492]		cell viability [A492]		cell viability [A492]		cell viability [A492]		cell viability [A492]	
Effector [µg/ml]												
10	0.982	1.069	1.024	1.023	0.893	0.912	0.977	0.939	1.175	0.982	0.964	0.992
2	0.934	1.01	0.983	0.95	0.932	0.997	0.959	0.944	1.005	0.938	0.945	1.037
0.4	0.937	0.91	0.973	0.918	0.986	0.925	0.948	0.949	0.98	0.965	1.044	0.919
0.08	0.894	0.882	0.91	0.905	0.914	0.949	0.977	0.926	0.985	0.901	1.102	0.888
0.016	0.875	0.906	0.855	1	0.9	0.935	0.967	0.955	0.966	0.976	1.029	0.983
0.0032	0.925	0.778	0.946	0.972	0.979	0.992	0.994	0.825	0.995	1.009	1.094	1.002
0.00064	0.938	1.013	0.92	0.896	0.979	0.995	1.035	1.087	1.046	1.096	1.053	0.876
0.000064	0.992	0.713	0.986	0.998	0.995	0.926	1.044	1.011	1.043	0.925	1.108	0.962

Quintas et al	Figure 5	Panel B										
Reporter cells:		Jurkat FcγRIIa NFAT-luc			Jurkat FcγRIIa NFAT-luc			Jurkat FcγRIIa NFAT-luc			Jurkat FcγRIIa NFAT-luc	
Effector:		Adalimumab			Atacicept			Belimumab			BCMA-Fc-739 not size-fractionated)	
Measure:		RLU signal			RLU signal			RLU signal			RLU signal	
Effector [µg/ml]												
1	-6320	-7170	1050	-5710	-12250	17080	-20	-2670	8050	88030	87680	90890
0.3333333	-1790	800	5770	-770	4780	21830	2880	-780	10720	88270	98460	103920
0.1111111	-220	9020	5860	-1610	8170	14270	2010	1830	11190	72860	94950	99680
0.03703704	-5610	-5870	7970	-4760	4680	10870	-1460	3560	8250	48880	68020	68860
0.01234568	-2260	-4710	7290	-4000	3000	7040	1820	2150	10320	36260	41010	34860
0.004115226	2590	-7750	12500	-1490	-590	9590	30	5660	-3900	12520	14250	20090
0.001371742	-2930	1170	10600	-4200	-4580	4540	2410	1250	3550	-130	11250	7650
Ø	0	0	0	0	0	0	0	0	0	0	0	0

Remark: Background signal in untreated cells (Ø) (~100'000) was subtracted
