# Supplementary material for: Inhibition of Membrane-Bound BAFF by the Anti-BAFF Antibody Belimumab
Source: Front Immunol. 2018 Nov 20;9:2698. doi: 10.3389/fimmu.2018.02698 (PMC6256835; doi:10.3389/fimmu.2018.02698)

FACS plots of Fig. 1A

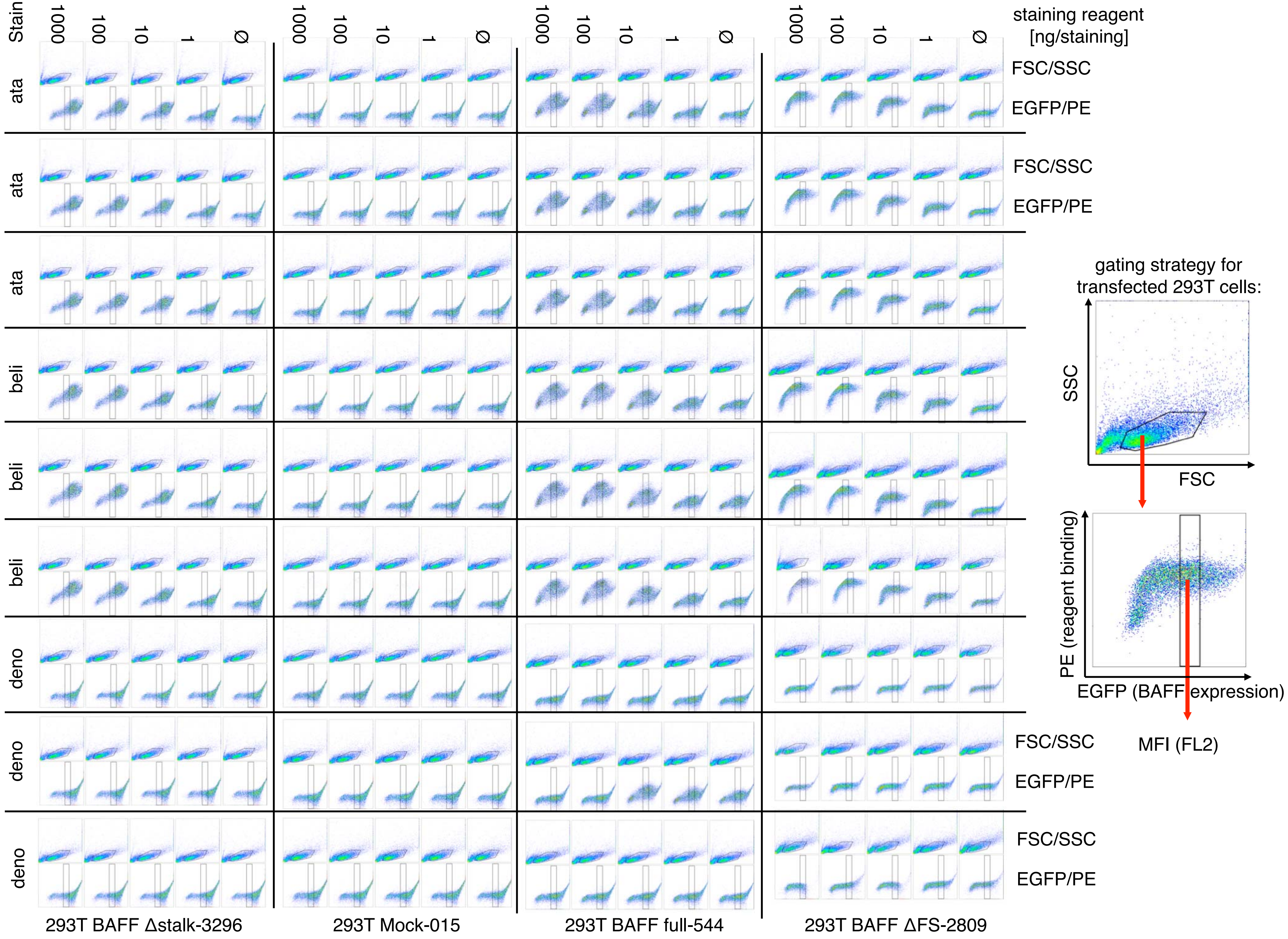

FACS plots of Fig. 1B

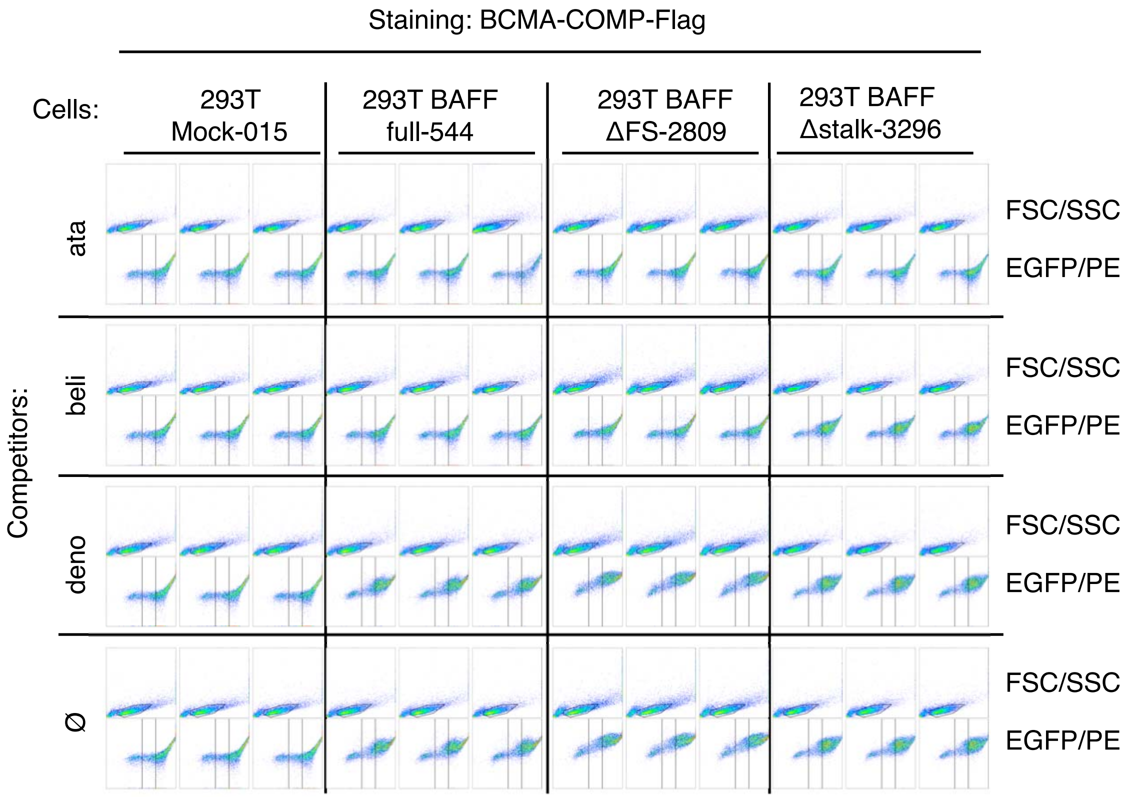

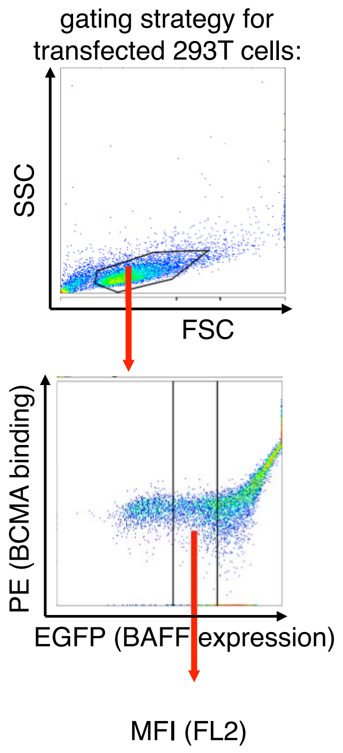

FACS plots for Fig. 2A

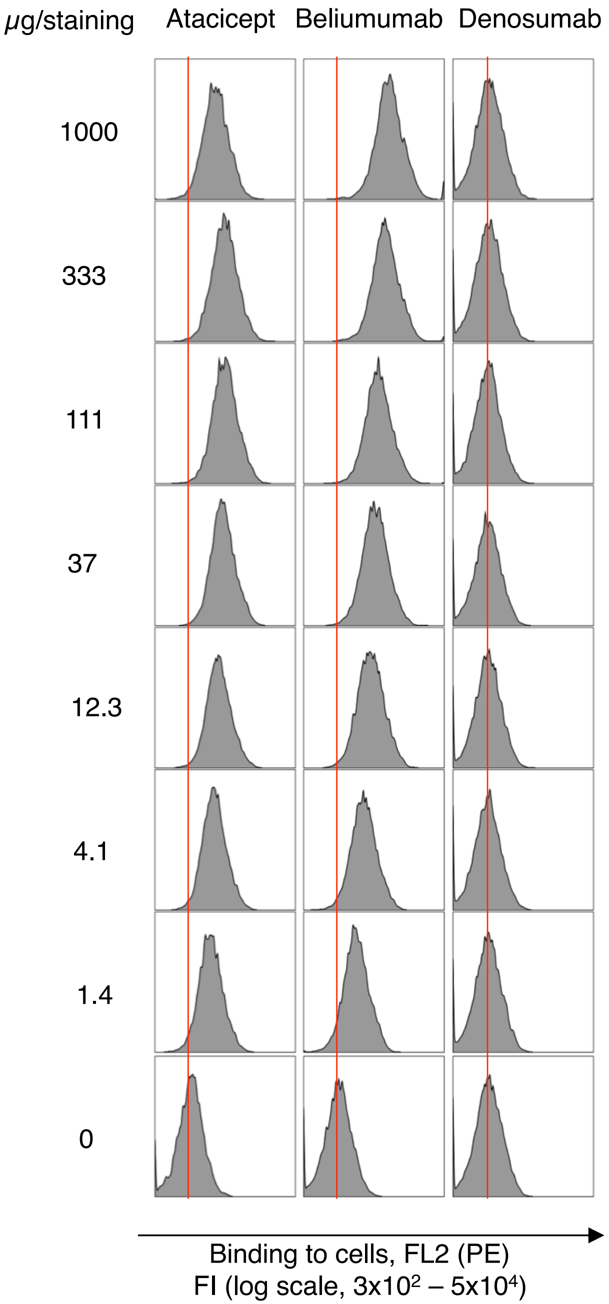

FACS plots for Fig 2C-F

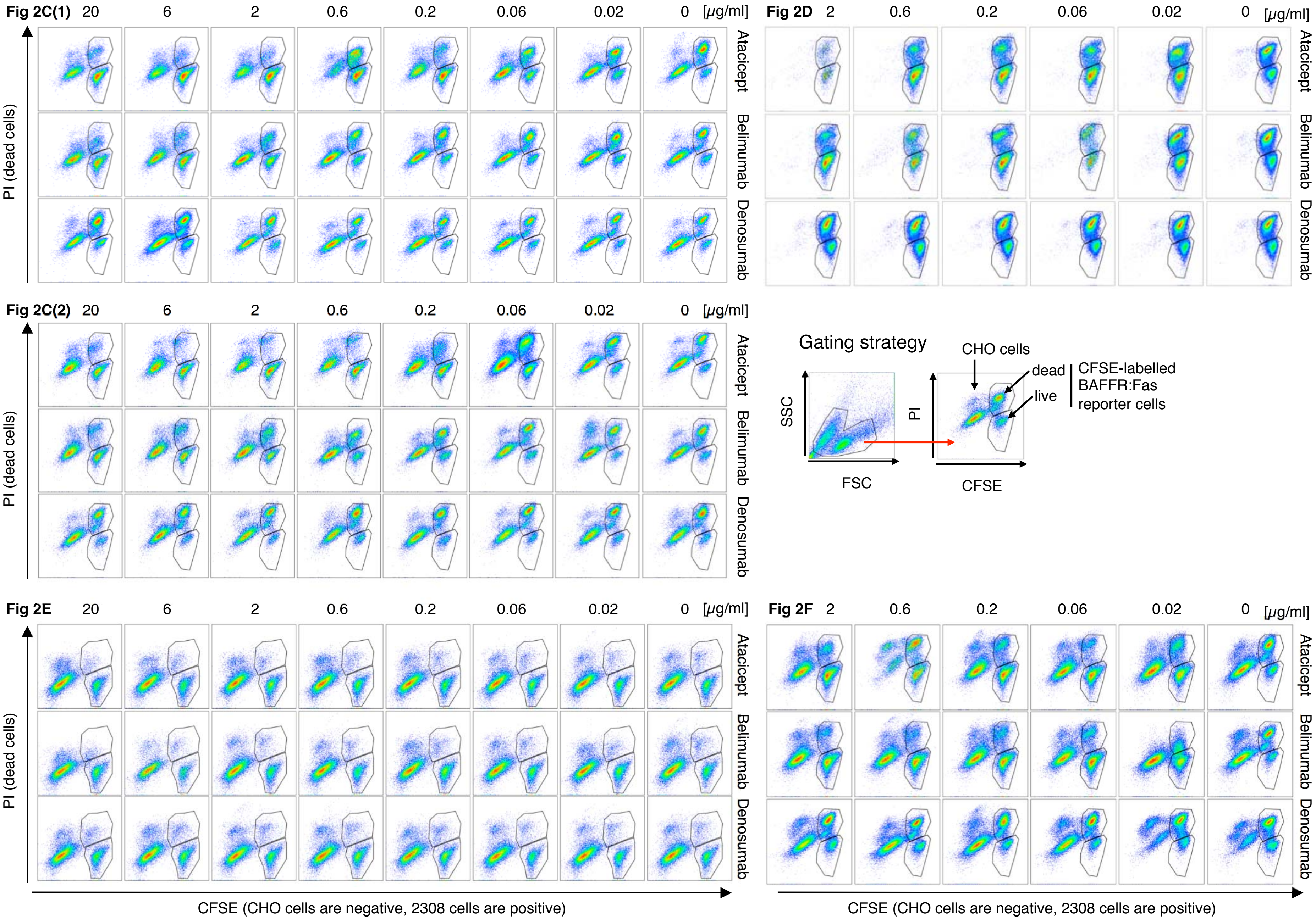

FACS plots for Fig 3A

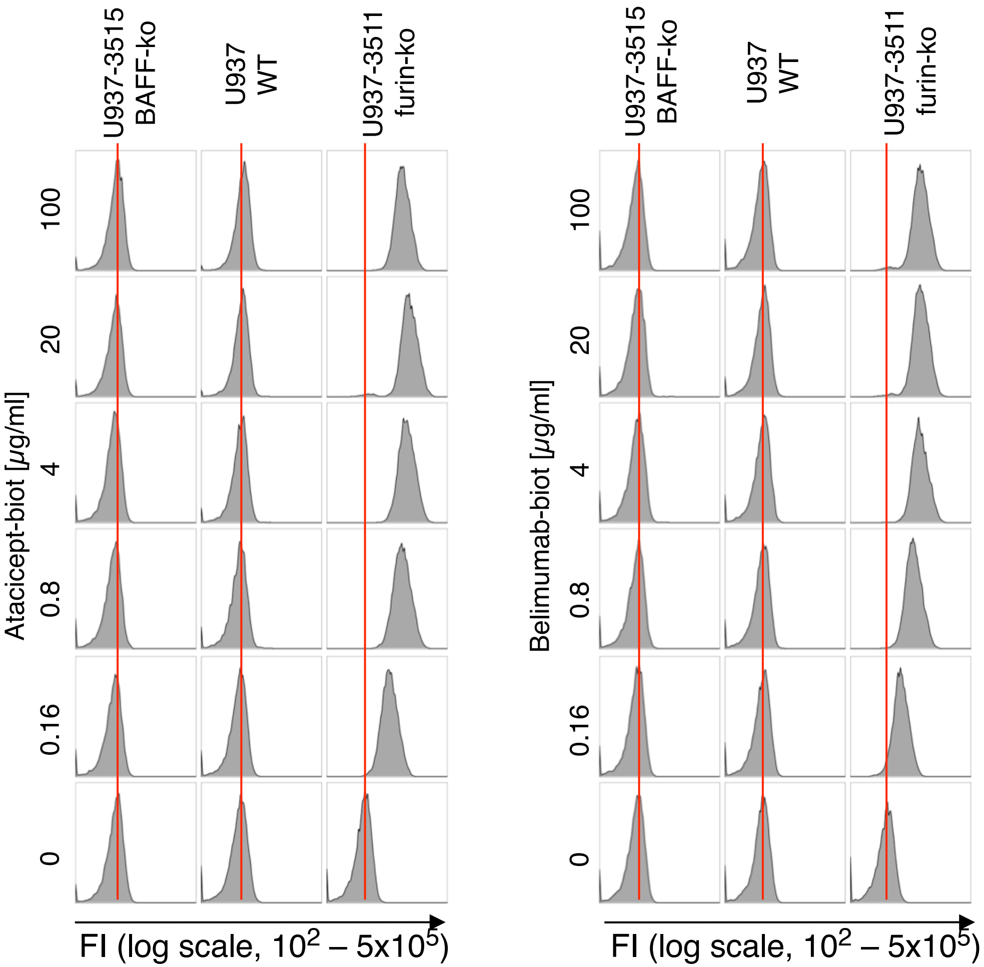

FACS plots of Fig. 3CDEF

Cytotoxic test with U937-furin-ko, Fc-BAFF and controls. Inhibition with atacicept, belimumab and control

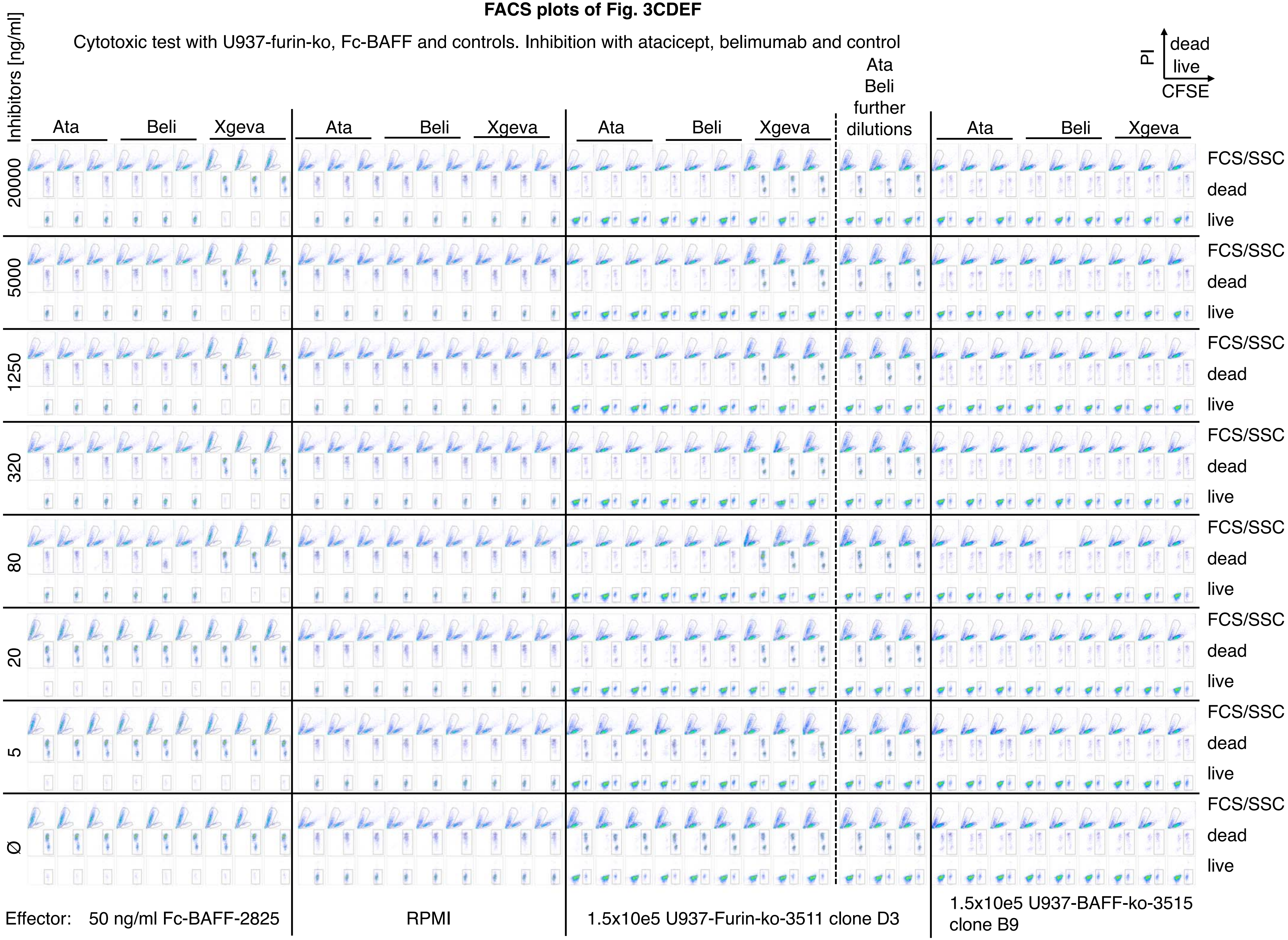

Supplement: Supplementary file 3 [file Data_Sheet_2.PDF]
